# Integrated multi-omics reveals methylation-linked biological heterogeneity in intermediate-risk pediatric acute myeloid leukemia

**DOI:** 10.1016/j.isci.2026.118028

**Published:** 2026-11-02

**Authors:** Harshita Makkar, Jagdish Prasad Meena, Riyaz Ahmad Mir, Aditya Kumar Gupta, Sameer Bakhshi, Anita Chopra, Pranay Tanwar, Rakhee Yadav, Lata Singh, Harsh Goel, Avanish Kumar Pandey, Pankaj Keshari, Sujata Bhattacharya, Ravi Kumar Majhi, Amitabh Singh, Shreshtha Kaushik, Sanjana Sarangarajan, Ganesh Kumar Viswanathan, Jasmita Dass, Deepam Pushpam, Rachna Seth

**Affiliations:** 1 Division of Pediatric Oncology, Department of Pediatrics, All India Institute of Medical Sciences, New Delhi, Delhi 110029, India; 2 Department of Biochemistry, All India Institute of Medical Sciences, New Delhi, Delhi 110029, India; 3 Department of Medical Oncology, Dr. B.R. Ambedkar Institute Rotary Cancer Hospital, All India Institute of Medical Sciences, New Delhi, Delhi 110029, India; 4 Laboratory Oncology Unit, Dr. B.R. Ambedkar Institute Rotary Cancer Hospital, All India Institute of Medical Sciences, New Delhi, Delhi 110029, India; 5 Department of Pediatrics, All India Institute of Medical Sciences, New Delhi, Delhi 110029, India; 6 Department of Pediatrics, Vardhman Mahavir Medical College and Safdarjung Hospital, New Delhi, Delhi 110029, India; 7 Department of Hematology, All India Institute of Medical Sciences, New Delhi, Delhi 110029, India

**Keywords:** pediatric acute myeloid leukemia, intermediate-risk AML, DNA methylation, epitypes, multi-omics integration, transcriptomics, pathway enrichment, immune activation

## Abstract

Intermediate-risk (IR) pediatric acute myeloid leukemia (AML) is clinically heterogeneous and lacks robust molecular stratification. We integrated DNA methylation, whole-exome, and transcriptome profiling in pediatric IR-AML. Unsupervised clustering of methylation profiles identified three stable epitypes (E1-E3) with distinct biological programs: E1 showed enrichment of stress-response and inflammatory pathways, E2 showed proliferative and survival signaling, and E3 showed transcriptional and developmental regulation with global hypomethylation. Despite heterogeneous mutational profiles, pathways converged across epitypes, indicating distinct genetic routes to shared functional outcomes. Immune activation was concordant across transcriptomic and epigenetic layers, with epitype-dependent variation in magnitude. Epitypes were associated with survival, with E1 showing poorer outcomes than E2 and E3. Projection onto an independent pediatric AML cohort showed partial conservation of epitype-associated methylation programs. These findings suggest that epigenetic stratification can capture clinically relevant heterogeneity beyond conventional genomic classification.

## Highlights

• DNA methylation identifies three distinct epitypes in intermediate-risk pediatric acute myeloid leukemia (AML)
• Integrated methylome, exome, and transcriptome analyses reveal distinct epitype programs
• Methylation epitypes capture heterogeneity beyond conventional clinical classification
• Multi-omics provides a framework for stratifying intermediate-risk pediatric AML

## Graphical abstract

**Figure:**
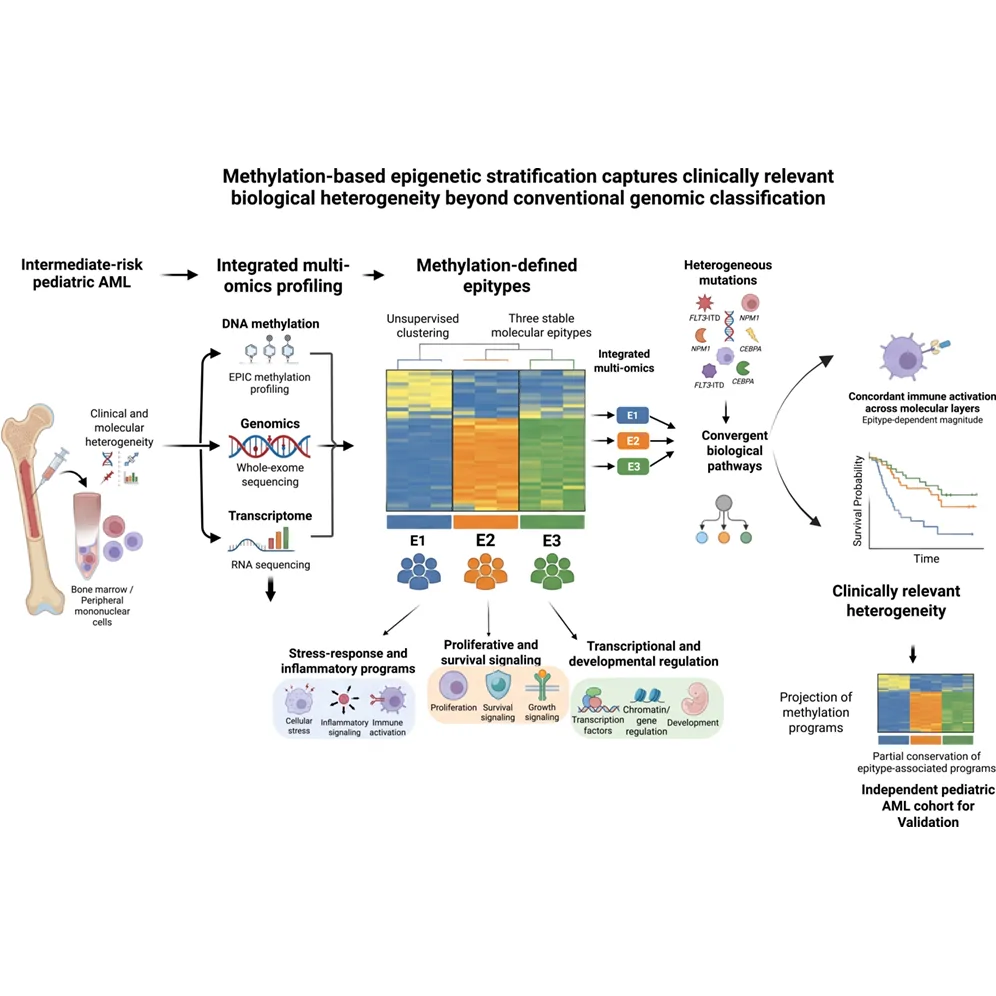

## Teaser

Immunology; Pediatrics; Disease; Omics; Genomics

## Introduction

Pediatric acute myeloid leukemia (AML) shows substantial biological heterogeneity and remains associated with significant morbidity and mortality despite advances in risk-adapted therapy and hematopoietic stem-cell transplantation.^1^^,^^2^^,^^3^^,^^4^ At present, risk stratification primarily relies on cytogenetic analysis, recurrent gene alterations, and measurable residual disease (MRD). However, intermediate-risk (IR) AML (IR-AML) is a clinically heterogeneous group including patients without distinctly favorable or adverse defining features, including many with cytogenetically normal disease, and is linked to inconsistent clinical outcomes.^5^^,^^6^ Improved molecular characterization of this subgroup is imperative to enhance prognostic evaluation and identify targetable biological factors.

In contrast to adult AML, childhood AML generally has a lower single-nucleotide mutational burden, characteristic combinations of cooperating genetic lesions, and a high frequency of fusion-driven subtypes. While genomic profiling has elucidated recurring alterations involving transcription factors, epigenetic regulators, and signaling pathways, this extensive array explains only part of the heterogeneity observed within IR pediatric AML, accentuating the necessity for integrated classifiers that transcend the limitations of single-marker risk evaluations.^5^^,^^7^

Epigenetic dysregulation, particularly involving promoter hypermethylation and enhancer remodeling, is instrumental in the pathogenesis of AML and reflects both the cellular origin and the oncogenic signaling environment.^7^^,^^8^^,^^9^ Methylation signatures have the capacity to delineate stable disease subgroups (“epitypes”) that possess prognostic and therapeutic significance extending beyond isolated mutations.^10^^,^^11^^,^^12^ Concurrently, transcriptomic profiling captures downstream functional diversity by revealing lineage priming, inflammatory signaling, metabolic reprogramming, and cell-cycle dynamics that differentiate molecular subtypes and elucidate potential therapeutic vulnerabilities.^13^^,^^14^^,^^15^ Concordance between methylation and expression signatures substantiates a model wherein the epigenetic landscape integrates upstream genetic events and microenvironmental influences to yield stable transcriptional identities.^16^^,^^17^^,^^18^^,^^19^

We hypothesized that integrating orthogonal molecular dimensions through systems-level pathway analysis would reconcile disparities among genotype, epigenetic state, and disease phenotype, addressing clinically pertinent heterogeneity in pediatric IR-AML. This study offers a conceptual and analytical framework for molecularly informed risk assignment and for prioritizing pathway-based interventions in this clinically intricate population.

In this research work, we performed an integrative multi-omic analysis of IR pediatric AML using genome-wide DNA methylation profiling, whole-exome sequencing, and RNA sequencing. In this study, we aimed to (i) define methylation-driven epitypes, (ii) evaluate their mutational landscapes, and (iii) identify convergent pathways and cellular programs underlying epitype-specific disease states.

## Results

### Study cohort and patient characteristics

We screened 76 pediatric AML patients (age <18 years), excluding 15 based on predefined criteria (including Down syndrome-associated AML, acute promyelocytic leukemia, other hematologic malignancies, prior treatment outside the study center, and insufficient sample). We collected peripheral blood or bone marrow samples from the remaining patients (*n* = 61). Cytogenetic analysis identified 20 patients (32.8%) with a normal karyotype (CN-AML). The integrative cohort included 13 IR-AML (ELN 2017) cases with available matched methylation and genomic data. Transcriptomic data were available for [*n* = 5] cases and [*n* = 3] controls.

### DNA methylation analysis identifies three epigenetic subgroups in intermediate-risk AML

Beta value density plots demonstrated high data quality following preprocessing and filtering, with characteristic bimodal distributions (β ≈ 0 and β ≈ 1) observed across all samples. Comparable density profiles between cases and controls indicated effective normalization and absence of major technical bias, supporting downstream differential methylation analyses (Figure 1A).

**Figure 1:**
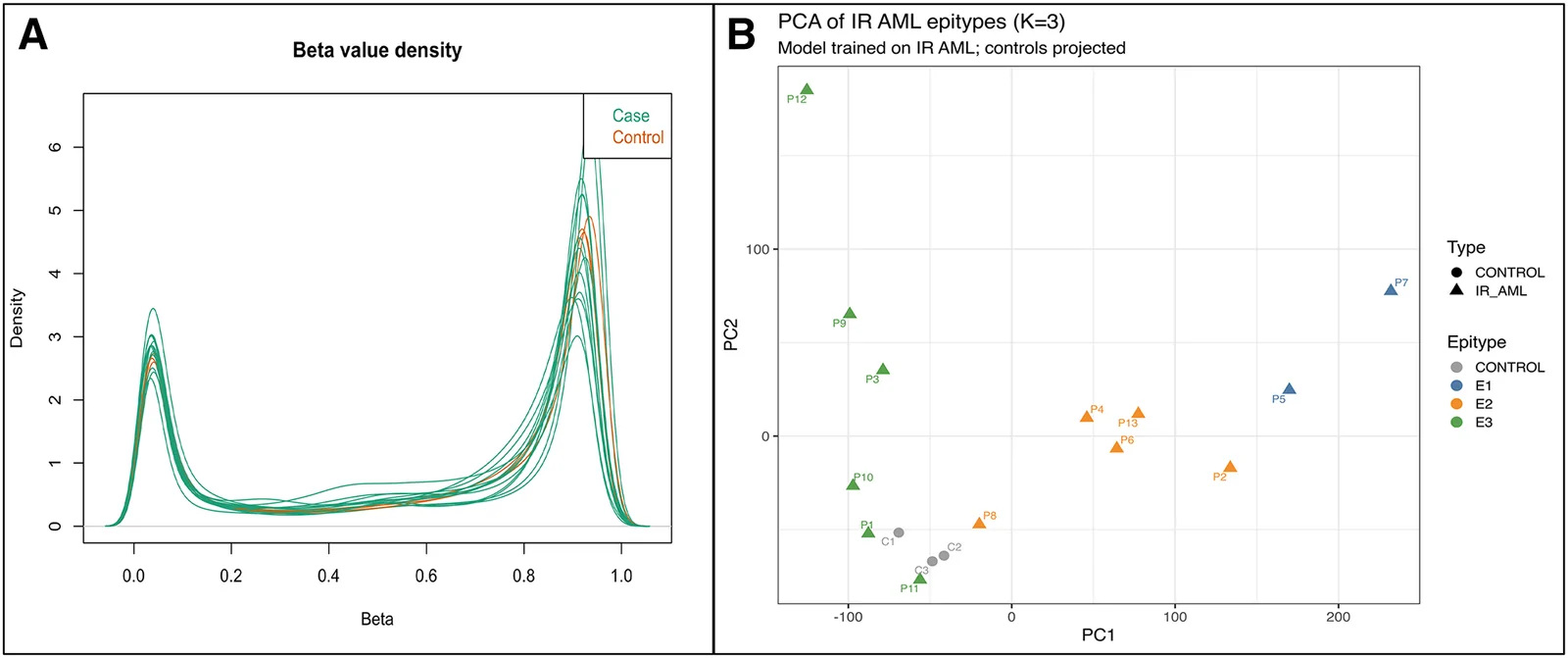
Methylation data quality and identification of three epitypes in intermediate-risk pediatric AML (A) Beta value density plots of case and control samples after preprocessing and filtering, demonstrating characteristic bimodal distributions (β ≈ 0 and β ≈ 1) consistent with high-quality methylation data. (B) Principal component analysis (PCA) of IR-AML samples identifying three methylation-defined epitypes (*K* = 3). Control samples were projected into the same PCA space. Each point represents a sample (IR-AML, triangles; controls, circles), colored by epitype (E1-E3), with controls shown in gray.

To quantitatively assess the robustness of methylation epitypes, we evaluated both cluster separation and stability using silhouette width and bootstrap-based best-match Jaccard similarity, respectively. Stability analyses were performed independently for promoter-restricted CpGs, CpG-island-proximal CpGs (island, shore, shelf), and whole-array variable CpGs across *K* = 2–6. For promoter-restricted CpGs, clustering solutions at *K* = 2 and *K* = 3 demonstrated the highest silhouette widths, with mean silhouette values of approximately 0.26 and 0.21, respectively. Increasing the number of clusters beyond *K* = 3 resulted in a marked decline in silhouette width (≤0.16 for *K* ≥ 4) and high stability (mean Jaccard >0.94) consistent with over-partitioning of the data. Notably, promoter-restricted and CpG-island-proximal epitype assignments were fully concordant at *K* = 3, confirming robustness of the identified structure across biologically related feature sets. These findings suggest that whole-array variable CpGs capture an alternative, less reproducible structure, likely influenced by broader epigenomic variability rather than promoter-centric regulatory methylation (Supplemental File 1 Validation of Epitypes).

Collectively, the combination of (i) maximal silhouette support at low *K*, (ii) high bootstrap stability (mean Jaccard >0.94) for promoter and CpG-island-proximal CpGs, and (iii) full concordance between these biologically relevant feature sets supports the selection of three stable methylation epitypes for downstream analyses (Figure 1B).

### Differential methylation and chromosomal distribution of significant CpGs

A volcano plot demonstrated widespread differential methylation between control and IR-AML samples, with widespread methylation differences observed across the genome, with top-ranked CpGs selected using combined statistical significance and methylation effect size (Figure S1A). Top-ranked CpGs were identified based on *p* < 0.05 and |Δβ|.

Genome-wide Manhattan analysis revealed broadly distributed differentially methylated CpGs across autosomes, with multiple loci exceeding the nominal significance threshold (*p* < 1 × 10^−5^) (Figure S1B). These signals span diverse functional classes, including lipid metabolism/one-carbon pathways (*CPT1A*, *LPCAT1*), TGF-β signaling (*ACVR1B*), ion transport (*KCNJ1*), hematopoietic/transcriptional regulation *(ETV6*, *HEY2*, *ZNF608*), and innate immune receptors (*CD180*), consistent with the biological processes identified in downstream methylation enrichment analyses.

### Unique CpG methylation identifiers of IR-AML epitypes

To define epigenetic heterogeneity within IR-AML, we identified epitype-associated CpG loci using an overall ANOVA (limma F-test) across methylation epitypes (E1-E3), which is consistent with the principal component analysis (PCA) (Figure 1B). The top 50 CpGs displayed highly structured and consistent methylation patterns that robustly segregated IR-AML epitypes and clearly distinguished leukemic samples from controls, revealing non-random methylation within pediatric IR-AML.

This suggests that epitype classification reflects coordinated epigenetic programs rather than stochastic CpG-level variation. Epitype-3 (E3) consistently exhibited lower β-values across a large fraction of CpGs, producing a broad hypomethylated block, whereas Epitype-2 (E2) displayed a predominantly hypermethylated profile (Figure 2). In contrast, E1 exhibited a distinctive bidirectional methylation pattern, with localized regions of both hypo- and hypermethylation, consistent with targeted regulatory remodeling rather than genome-wide methylation shifts.

**Figure 2:**
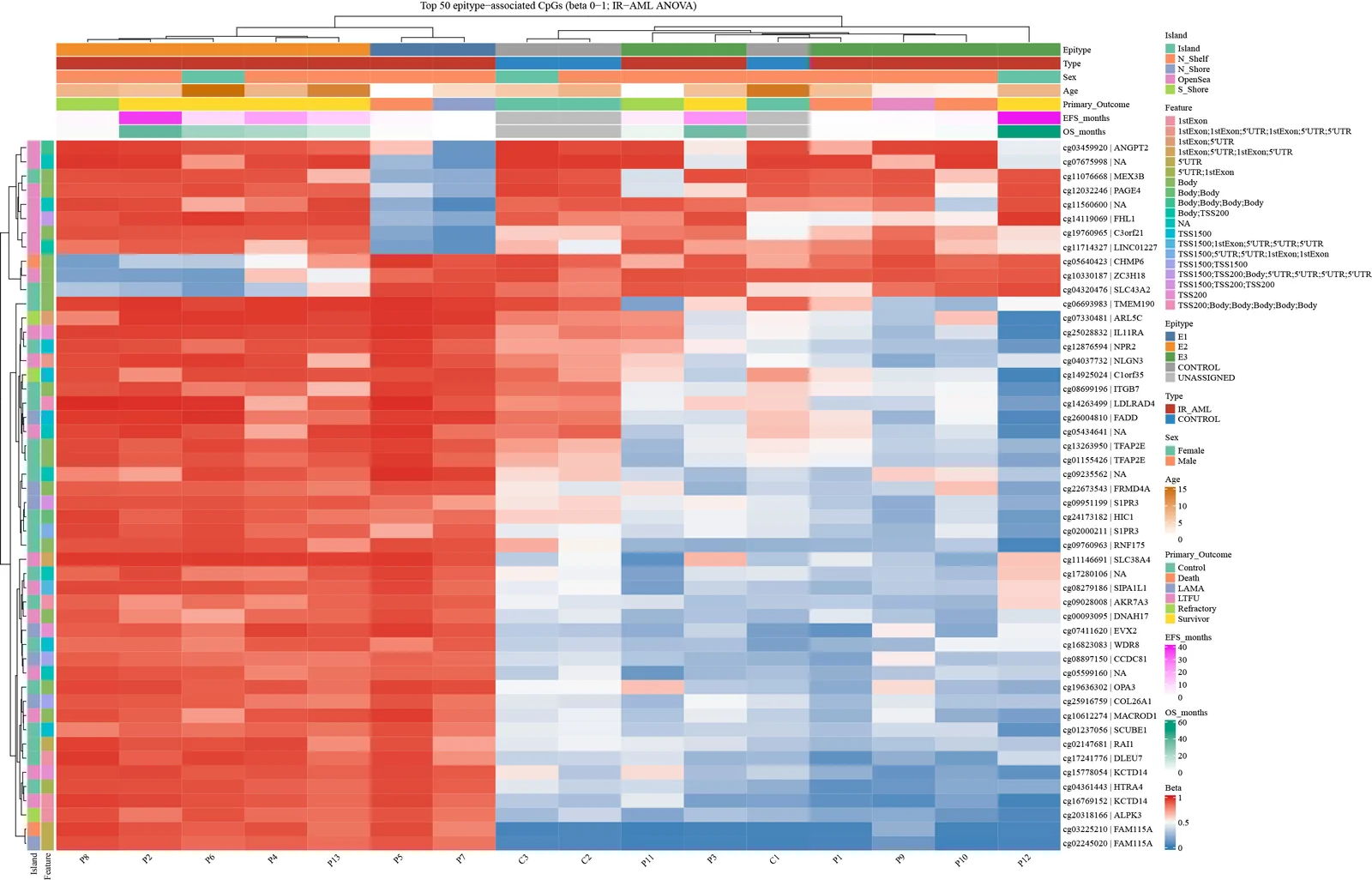
Heatmap of the top 50 epitype-associated CpGs in intermediate-risk AML (IR-AML), identified by ANOVA across epitypes (E1-E3) Rows represent CpGs (annotated as CpG|Gene) and columns represent samples, with β-values (0-1) indicating methylation levels. Unsupervised clustering reveals distinct epitype-specific methylation patterns, particularly separating Epitype-1 from E2 and E3. Row annotations denote CpG island context and genomic features.

Mapping CpGs to their corresponding genes revealed that multiple CpGs targeting the same genes followed concordant methylation behavior within E1, indicating gene-level biological coherence. Genes including *ANGPT2*, *PAGE4*, *MEX3B*, *FHL1*, *C3orf21*, and *LINC01227* consistently had E1-specific hypomethylation, while *FAM115A* and *KCTD14* showed E1-specific hypermethylation across independent CpGs. Notably, many informative CpGs mapped to non-island and gene-body regions, suggesting involvement of distal regulatory elements. Ranking CpGs by statistical significance and effect size (Δβ) demonstrated that CpGs contributing most strongly to the epitype also drove visual clusters, confirming their discriminative value_ (Figure S2). Representative loci comprised *S1PR3* and *ITGB7* (hypomethylated in E3) and *TFAP2E* and *RAI1* (hypermethylated in E2), further supporting epitype-specific methylation programs (Figure 2).

To characterize biological processes associated with differential DNA methylation in IR-AML, we performed pathway enrichment analysis using fgsea on promoter-associated methylation statistics (IR-AML vs. control). Gene Ontology Biological Process analysis identified a strong enrichment of developmental and neurogenic programs among the top differentially methylated processes, including neuronal differentiation, pattern specification, and embryonic morphogenesis, consistent with preferential hypermethylation of lineage-regulatory genes (Figure 3A). Reactome analysis showed a bidirectional enrichment pattern (Figure 3B): hypermethylation-associated pathways were enriched for G protein-coupled receptor (GPCR) signaling and developmental processes, whereas hypomethylation-associated pathways were linked to cell-cycle regulation, chromatin organization, RNA metabolism, Rho GTPase signaling, and immune pathways, including interleukin signaling and neutrophil degranulation.

**Figure 3:**
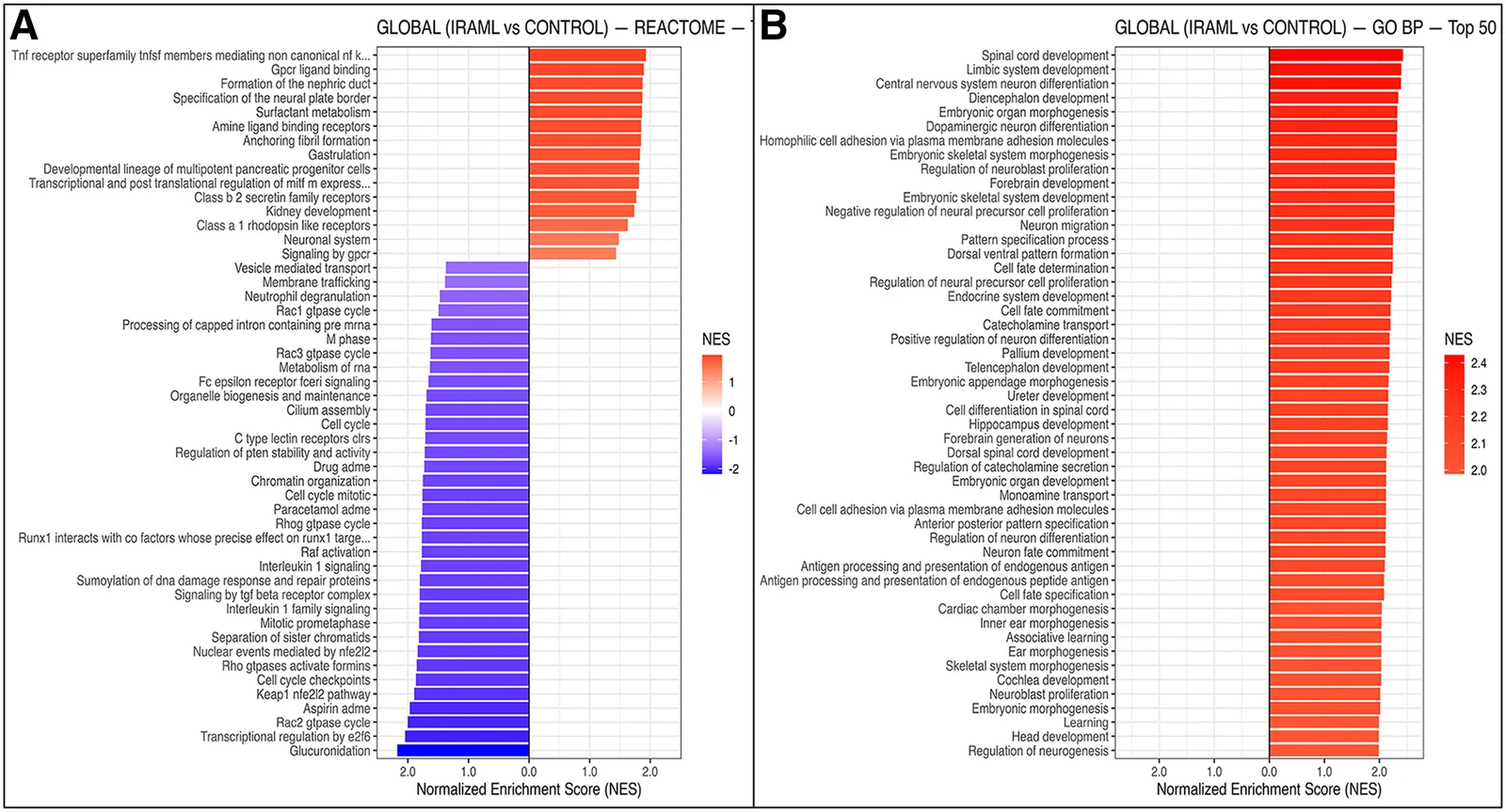
Global functional and pathway enrichment associated with differential DNA methylation in intermediate-risk AML (A and B) GO Biological Process and (B) Reactome pathway enrichment based on promoter-associated CpG methylation differences (IR-AML vs. control). Gene sets were ranked using *t*-statistics and analyzed by fgsea. Bars represent normalized enrichment scores (NES), with positive and negative values indicating relative hyper- and hypomethylation, respectively.

### Mutational landscape of key driver genes in intermediate-risk AML

Somatic variant analysis in recurrent AML driver genes revealed substantial inter-patient heterogeneity within the IR-AML cohort. The oncoplot highlights diverse driver gene alteration combinations, with no single mutation present across all cases. Some patients carried multiple driver mutations, while others exhibited a lower overall mutational burden (Figure S3A). Nucleotide substitution profiling indicated a transition-dominant pattern, with transitions occurring more often than transversions, in line with established mutational signatures in AML (Figure S3B).

In IR-AML, the mutational landscape was marked by pronounced inter-patient variability, with recurrent alterations affecting multiple biological pathways. Tumor mutational burden was generally low to moderate; there was no obvious enrichment across methylation epitypes. Signaling pathways were prominently affected, with alterations affecting *FLT3*, as well as recurrent alterations in *NOTCH1*, *KIT*, *JAK2*, and *CSF3R*, consistent with impaired RTK/JAK/NOTCH signaling (Figure S4).

Transcriptional regulators, including *GATA2* and *RUNX1*, were commonly mutated. Monoallelic *CEBPA* alterations were observed, including both in-frame and multi-hit events. Recurrent mutations were also observed in epigenetic modifiers (*TET2*, *IDH1)*, RNA splicing genes (*ZRSR2*), and cohesin complex components (*SMC1A*, *SMC3*), suggesting cooperative pathogenic processes. Additional alterations in *TP53* and *ATM* further highlighted disruption of apoptotic and DNA damage response pathways. Missense variants were the predominant variant class, with relatively fewer indels. We retained variants using relaxed inclusion criteria, including variants of uncertain significance, to support comprehensive pathway-level analysis.

Global fgsea based on mutation-derived gene-level statistics identified bidirectional pathway perturbations across Kyoto Encyclopedia of Genes and Genomes (KEGG) and Gene Ontology (GO) Biological Process collections (Figure 4). KEGG analysis showed enrichment of key signaling pathways, including MAPK, JAK-STAT, Wnt, PI3K-AKT, and GPCR signaling, along with immune and metabolic processes. In contrast, negatively enriched pathways were predominantly associated with cell-cycle regulation, DNA replication, ubiquitin-mediated proteolysis, and p53 signaling, indicating disruption of proliferative control mechanisms. Consistently, GO BP analysis revealed enrichment of intracellular transport, cytoskeletal organization, vesicle-mediated trafficking, and metabolic processes, whereas negatively enriched terms were linked to cell-cycle progression, chromatin organization, and stress-response pathways. Together, these concordant bidirectional patterns indicate mutation-associated dysregulation of core signaling, metabolic, and cell-cycle programs in IR-AML (Figures 4 and 5A).

**Figure 4:**
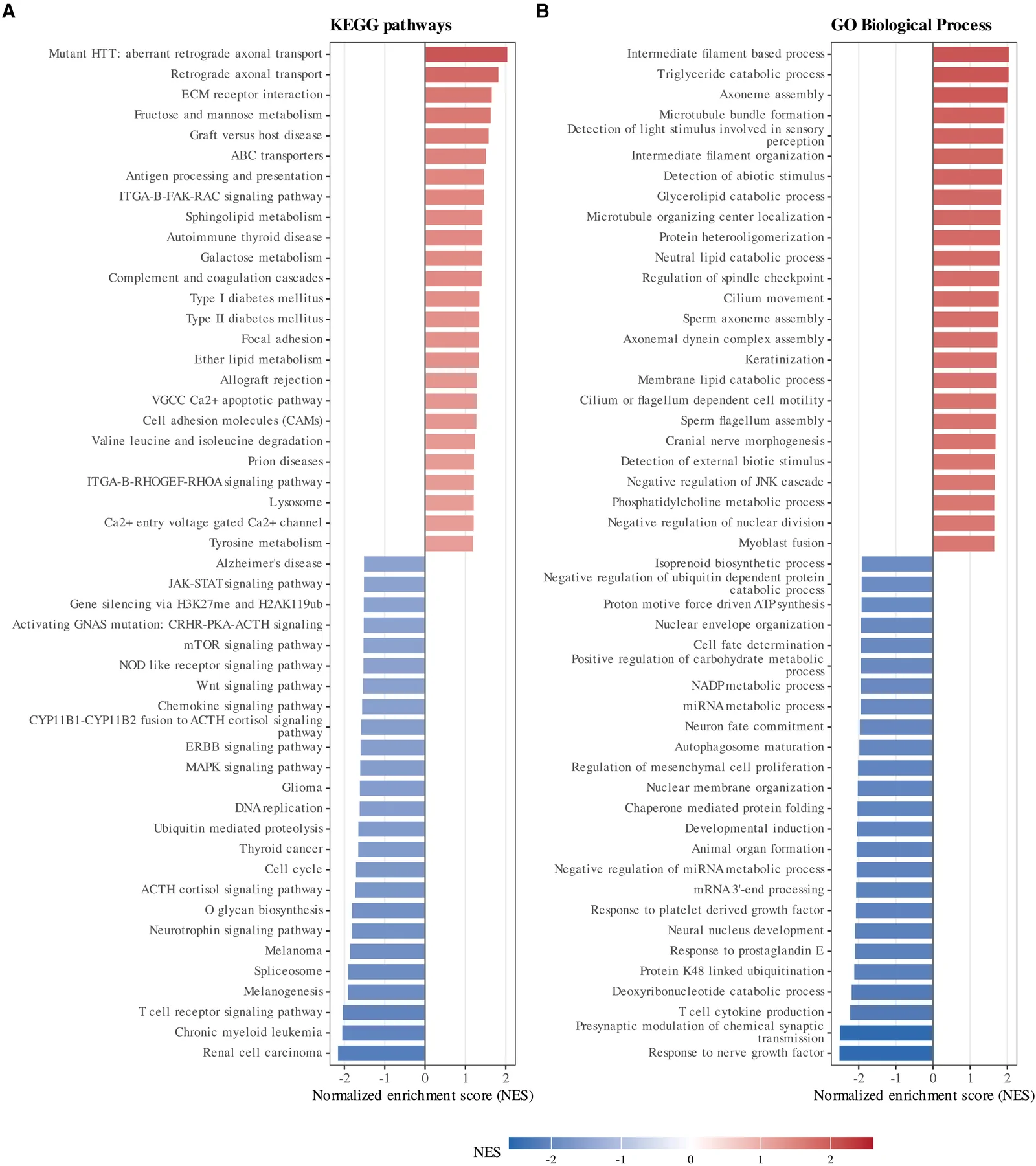
Pathway enrichment analysis based on mutation-derived gene-level statistics in intermediate-risk AML Bar plots showing the top 35 enriched pathways identified by fgsea. (A) KEGG pathways; (B) Gene Ontology Biological Process (GO BP) terms. Pathways are ordered by adjusted *p*-value and normalized enrichment score (NES). Positive and negative NES indicate the direction of enrichment, with color intensity reflecting magnitude.

**Figure 5:**
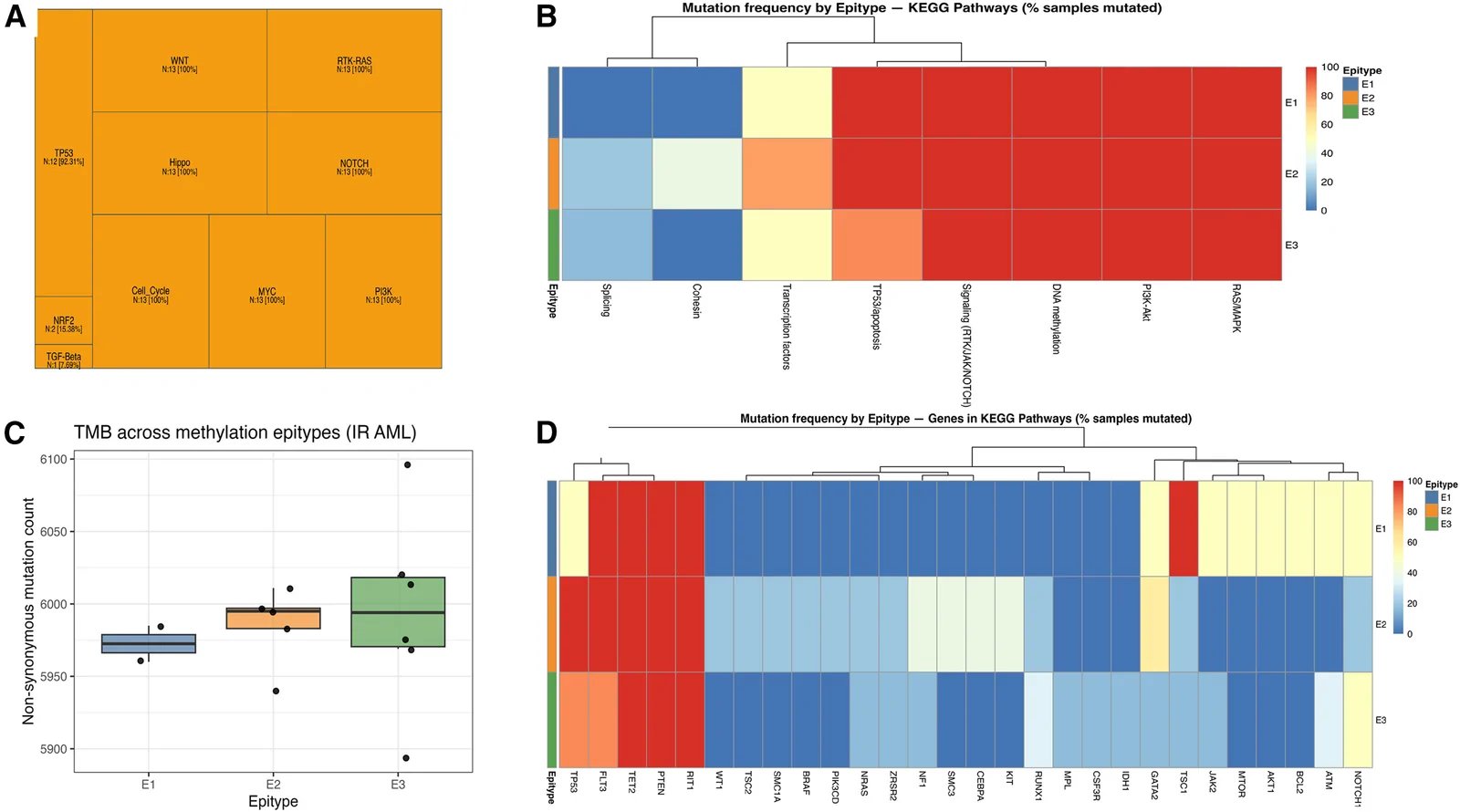
Epitype-resolved pathway-level mutational architecture in intermediate-risk AML (A) Treemap showing the distribution of somatic mutations across KEGG pathways in the full cohort, with box size representing the proportion of samples affected. (B) Bubble plots of the top 20 enriched KEGG pathways based on mutation-derived gene-level statistics for the overall cohort and for each epitype (E1-E3 vs. others). Bubble color indicates normalized enrichment score (NES), and size reflects gene set size. (C) Heatmap of pathway-level mutation frequencies (%) stratified by epitype. (D) Gene-level heatmap showing mutation frequencies across KEGG pathways by epitype.

### Mutation and integrative pathway analysis reveal epitype-specific convergent biological programs in intermediate-risk AML

Stratification by methylation-defined epitypes revealed distinct patterns of pathway involvement. Epitype 1 (E1) showed preferential enrichment of TP53-associated and apoptosis-related pathways, along with stress-response and inflammatory signaling, consistent with a biologically dysregulated phenotype. Epitype 2 (E2) was characterized by enrichment of proliferative and survival pathways, including RTK-RAS, PI3K-AKT, and cell-cycle signaling, indicating a growth-driven molecular program. In contrast, Epitype 3 (E3) demonstrated enrichment of transcriptional regulation, chromatin organization, and NOTCH signaling pathways, reflecting a distinct regulatory and developmental state (Figure 5D).

Comparison of mutation-derived and methylation-based pathway enrichment profiles further demonstrated that epitype separation is reinforced at the pathway level by both genetic and epigenetic mechanisms. While some pathways, such as PI3K-AKT and cell-cycle signaling, were enriched across both layers in specific epitypes, others appeared preferentially driven by either mutational or epigenetic alterations. Notably, pathways including ECM-receptor interaction, ABC transporters, and metabolic processes showed consistent enrichment across epitypes, albeit with variation in direction and magnitude (Figures 6A and 6B).

**Figure 6:**
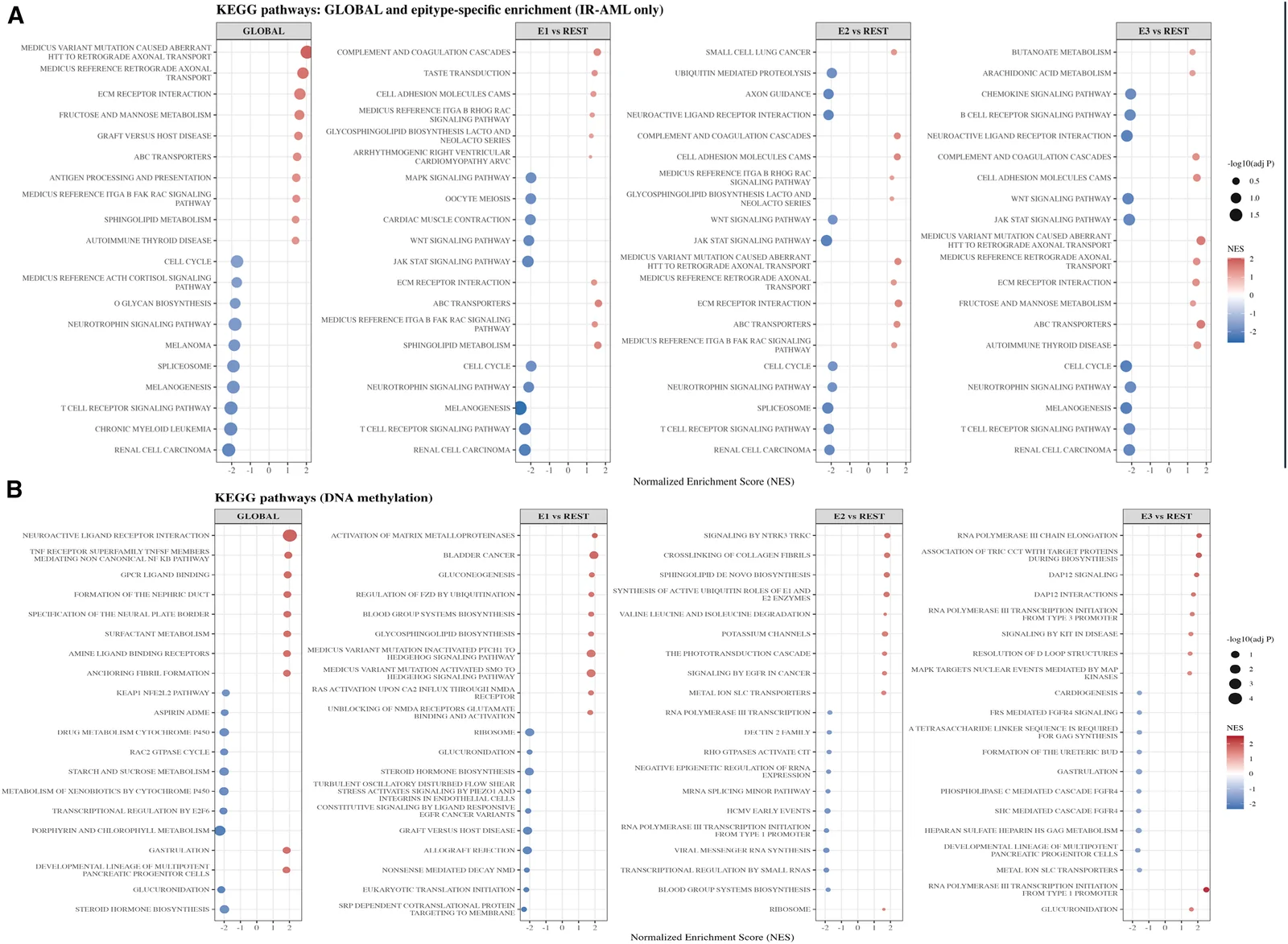
Integrated mutation- and methylation-based pathway enrichment across epitypes in intermediate-risk AML (A) Bubble plots showing KEGG pathway enrichment from mutation-based FGSEA for the global cohort and epitype-specific comparisons (E1-E3 vs. REST). (B) Bubble plots showing KEGG pathway enrichment from methylation-based FGSEA for the same comparisons. Pathways are ordered by normalized enrichment score (NES). Bubble color indicates direction of enrichment (red, positive; blue, negative), and size reflects −log10 adjusted *p* value. Together, panels (A) and (B) highlight concordant and divergent pathway perturbations across epitypes driven by genetic and epigenetic mechanisms.

Collectively, these findings indicate that IR-AML is characterized by epitype-specific convergence of genetic and epigenetic alterations onto shared biological programs, including immune activation, proliferative signaling, stress response, and developmental regulation. This pathway-centric organization provides a unifying framework for understanding molecular heterogeneity within IR-AML.

### Exploratory transcriptomic analysis reveals distinct expression profiles between pediatric AML and controls

PCA of variance-stabilized expression data demonstrated clear separation between pediatric AML samples and controls. The first two principal components accounted for a substantial proportion of the variance (PC1: 28.2%, PC2: 19.4%), indicating global transcriptional differences between the two groups (Figure S5A). Differential expression analysis identified a subset of consistently dysregulated genes (Figure S5B). Using an exploratory threshold of FDR <0.20, both upregulated and downregulated transcripts were observed. Genes such as *RGMA*, *ADCY9*, *KCNE5*, and *P2RY2* were upregulated, whereas *EXOC3L2* and *ITGA2B* were among the downregulated genes.

Gene set enrichment analysis using ranked differential expression identified distinct pathway-level alterations between pediatric AML and controls (Figure 7). Biological process enrichment revealed strong upregulation of immune-related pathways, including antigen processing and presentation, leukocyte chemotaxis, dendritic cell migration, and interleukin-1 production. In contrast, pathways related to RNA processing, mRNA splicing, protein localization, and developmental processes were relatively downregulated, indicating suppression of core transcriptional and cellular regulatory mechanisms (Figure 7B). Correspondingly, KEGG pathway analysis showed enrichment of cytokine signaling, chemokine signaling, and immune-related pathways, alongside downregulation of proteasome, spliceosome, and metabolic pathways (Figure 7A). This analysis highlights that pediatric AML is characterized by coordinated immune activation coupled with suppression of fundamental transcriptional and proteostatic processes, reflecting widespread transcriptomic reprogramming.

**Figure 7:**
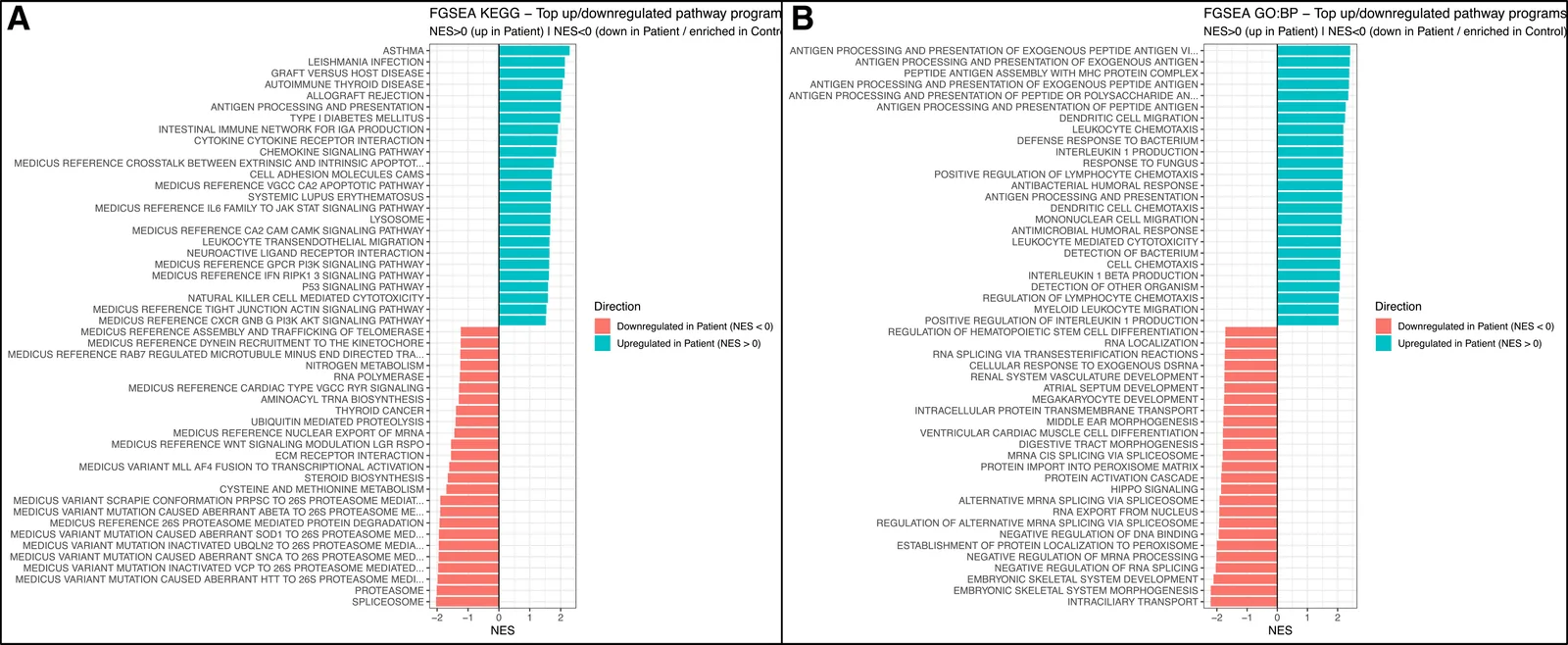
Transcriptome-based pathway enrichment in pediatric AML FGSEA showing the top enriched pathways. (A) Gene Ontology Biological Process terms highlighting enrichment of immune-related pathways in AML and downregulation of RNA processing and developmental programs. (B) KEGG pathway enrichment showing activation of cytokine and chemokine signaling, with suppression of proteasome and spliceosome pathways. Positive and negative NESs indicate enrichment in AML and controls, respectively.

### Integrated pathway analysis reveals concordant immune activation and regulatory suppression across transcriptomic and epigenetic layers

Comparative pathway enrichment analysis integrating transcriptomic and methylation data in IR pediatric AML revealed concordant, biologically coherent alterations across molecular layers. RNA-seq-based FGSEA revealed enrichment of immune and inflammatory pathways, including antigen presentation, cytokine-receptor interaction, and chemokine signaling, suggesting active immune transcriptional programs. In contrast, pathways related to RNA processing, spliceosome function, and proteasomal degradation were comparatively downregulated. Methylation-based analysis demonstrated broader pathway perturbations, with enrichment of overlapping immune pathways, which supports concordance between transcriptional activation and epigenetic regulation. Additional enrichment of developmental and signaling pathways reflects extensive epigenetic remodeling. Immune pathway activation was observed across the cohort but varied by epitype, with stronger enrichment in specific subgroups (e.g., E1). Collectively, these findings highlight immune activation as a shared feature of IR pediatric AML, supported by both transcriptional and epigenetic mechanisms, with epitype-dependent variation (Figures 7 and 8).

**Figure 8:**
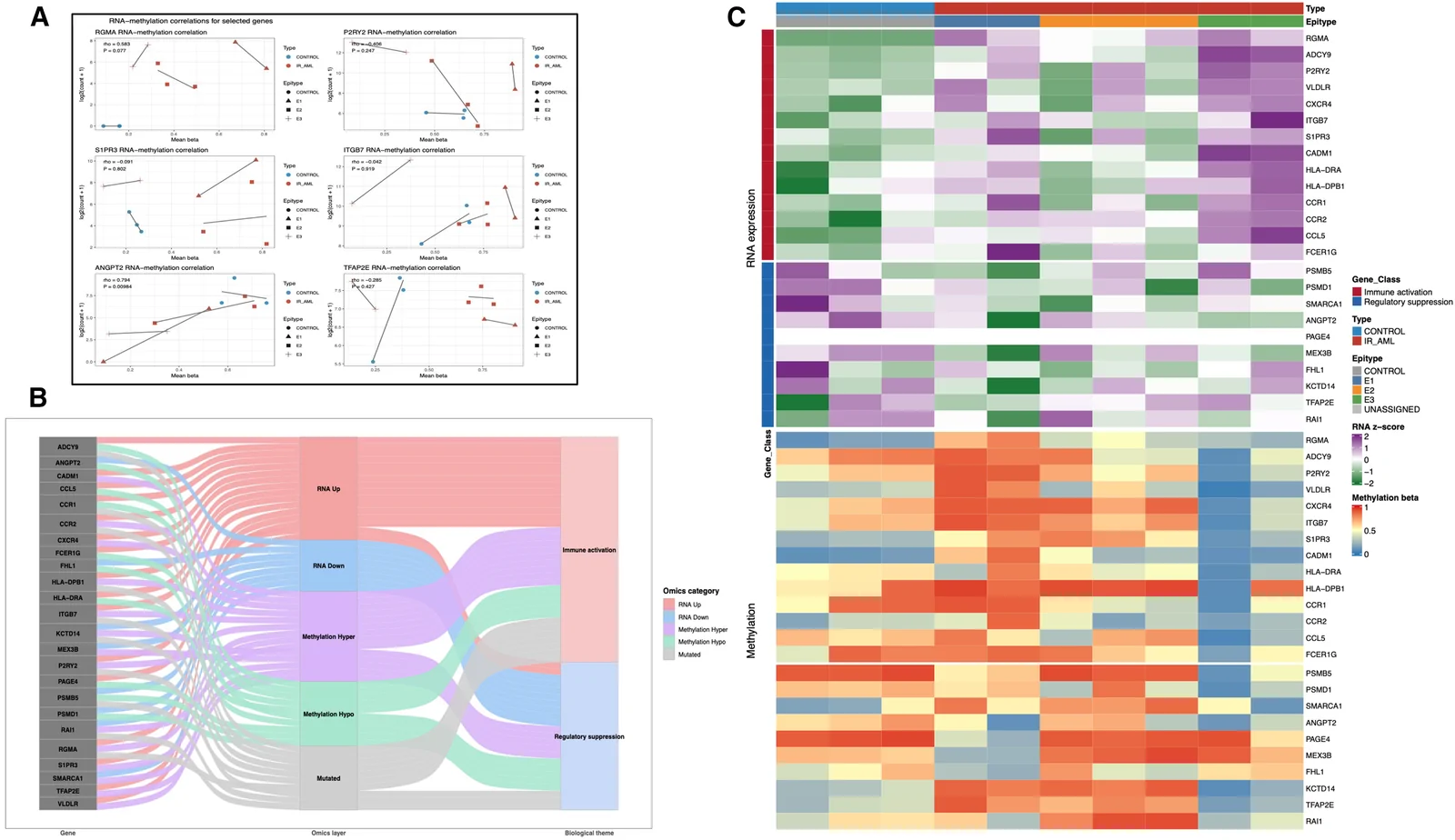
Multi-omics integration of RNA expression, DNA methylation, and mutation patterns in pediatric AML (A) Scatterplots showing the relationship between DNA methylation (mean β-value) and RNA expression (log2-transformed counts) for selected genes. Points represent samples, colored by disease status and shaped by epitype; Spearman correlation coefficients (ρ) and *p* values are indicated. (B) Alluvial plot summarizing gene-level multi-omics integration, linking RNA expression, methylation status, and mutation categories to functional classes. (C) Integrative heatmap of RNA expression (*Z* score; upper panel) and DNA methylation (β-value; lower panel) across selected genes, with samples annotated by disease type and epitype.

To further dissect the relationship between transcriptional and epigenetic alterations underlying the integrated pathway-level signatures (Figure 8), we examined gene-level associations between RNA expression and DNA methylation. Integrated gene-level analysis revealed heterogeneous regulatory relationships, with both concordant and discordant expression-methylation patterns, indicating that epigenetic and transcriptional alterations are not uniformly coupled and that pathway-level convergence arises from diverse gene-specific regulatory mechanisms. Correlation analysis between gene expression and DNA methylation revealed predominantly inverse relationships consistent with epigenetic regulation. However, both concordant and discordant patterns were observed, indicating heterogeneous coupling between methylation and transcription across genes and epitypes. Notably, genes such as *FGFR1OP2*, *AKAP1*, and *MRPL1* exhibited strong negative correlations, whereas *PCIF1* showed a positive association, suggesting context-dependent regulatory mechanisms (Figure S6).

Gene-wise correlation analysis further supported this heterogeneity. Most genes, including *S1PR3*, *ITGB7*, *P2RY2*, and *TFAP2E*, showed weak or non-significant associations, whereas *RGMA* demonstrated a modest positive trend. In contrast, *ANGPT2* exhibited a strong positive correlation between methylation and expression (ρ = 0.79, *p* < 0.01), suggesting context-dependent regulatory effects (Figure 8A). Notably, *VCAM1* showed a robust negative association, supporting regulation of immune-related transcriptional programs (Figure S6). Alluvial integration further highlighted heterogeneous regulatory trajectories, with both concordant and discordant RNA-methylation patterns across genes (Figure 8B).

### Epitype-specific survival differences in intermediate-risk pediatric AML

Kaplan-Meier analysis showed differences in event-free survival (EFS) and overall survival (OS) across methylation-defined epitypes in IR pediatric AML (log rank *p* = 0.0094 for both endpoints). Epitype 1 (E1) was associated with poorer survival, with earlier events and reduced survival probabilities compared with Epitypes 2 (E2) and 3 (E3), which showed relatively improved outcomes. Pairwise comparisons indicated a higher risk of adverse outcomes in E1 relative to E2 and E3, whereas differences between E2 and E3 were less pronounced. Hazard ratio estimates showed wide confidence intervals, reflecting the limited sample size. Nonetheless, the reproducible separation of survival curves across both EFS and OS suggests that methylation-defined epitypes capture clinically meaningful heterogeneity within IR-AML (Figure 9).

**Figure 9:**
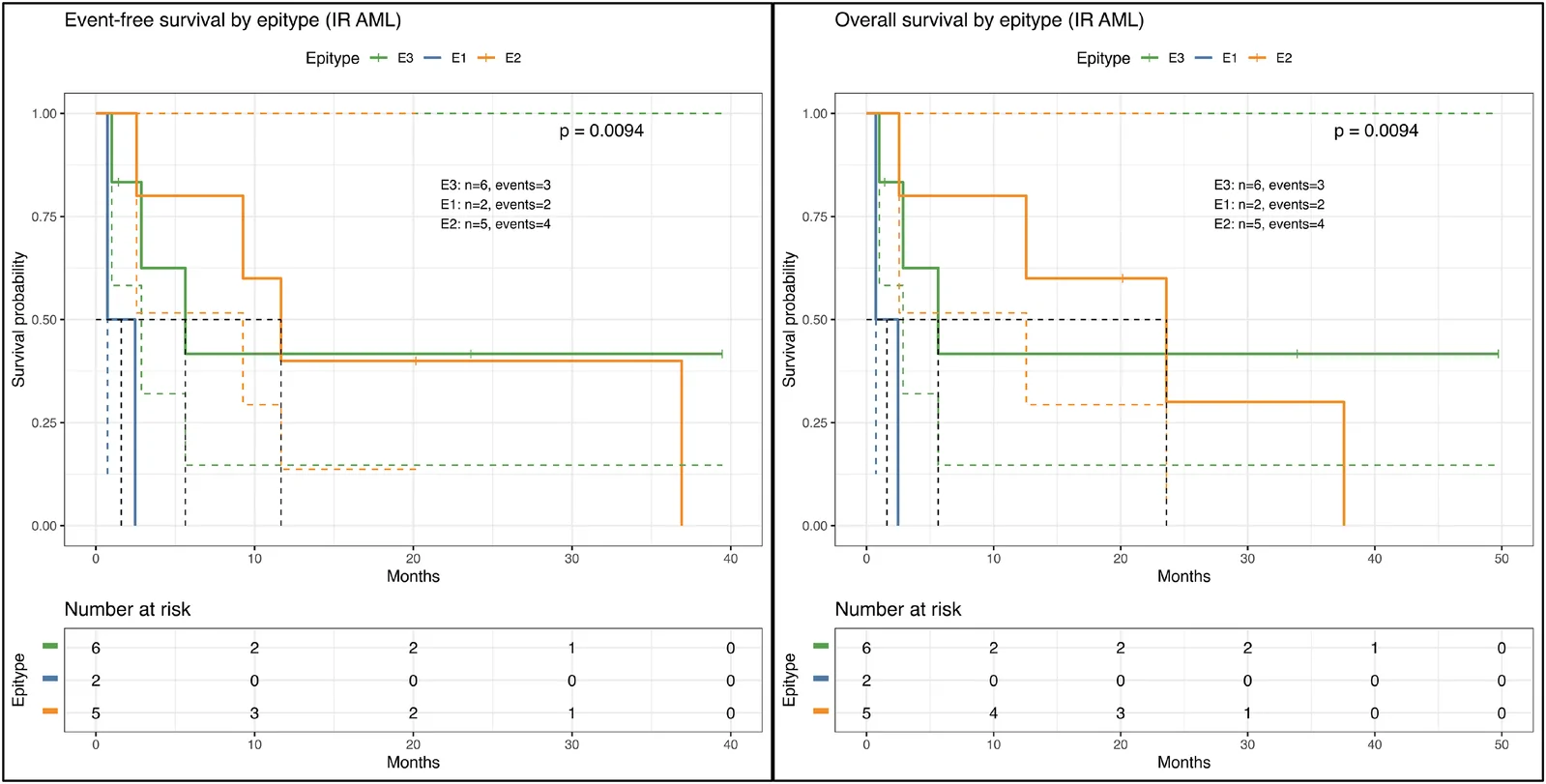
Kaplan-Meier survival analysis of methylation-defined epitypes in intermediate-risk pediatric AML (A and B) Event-free survival and (B) overall survival stratified by epitypes (E1-E3). Survival probabilities are shown over time (months), with numbers at risk indicated below each panel. Survival differed across epitypes (log rank *p* = 0.0094 for both endpoints). E1 was associated with poorer survival compared with E2 and E3, whereas E2 and E3 showed relatively improved outcomes. Pairwise hazard ratios are shown; wide confidence intervals reflect the limited sample size.

### External validation and projection-based reproducibility of epitypes

Projection-based validation using an independent pediatric AML methylation cohort (GSE133986) demonstrated partial but consistent correspondence with the epitype framework established in the discovery cohort. Without re-clustering, external samples were assigned to E1-E3 centroids through a correlation-based approach, enabling a direct assessment of epitype reproducibility. PCA indicated that the projected samples occupied the same global methylation space as discovery samples, albeit with greater dispersion, which likely reflects the inclusion of a broader AML population beyond IR disease (Figure 10A). All three epitype states were represented; however, E1-like samples were less frequent and displayed comparatively lower assignment confidence. Projection-based validation assesses the reproducibility of biological structure rather than the replication of clustering.

**Figure 10:**
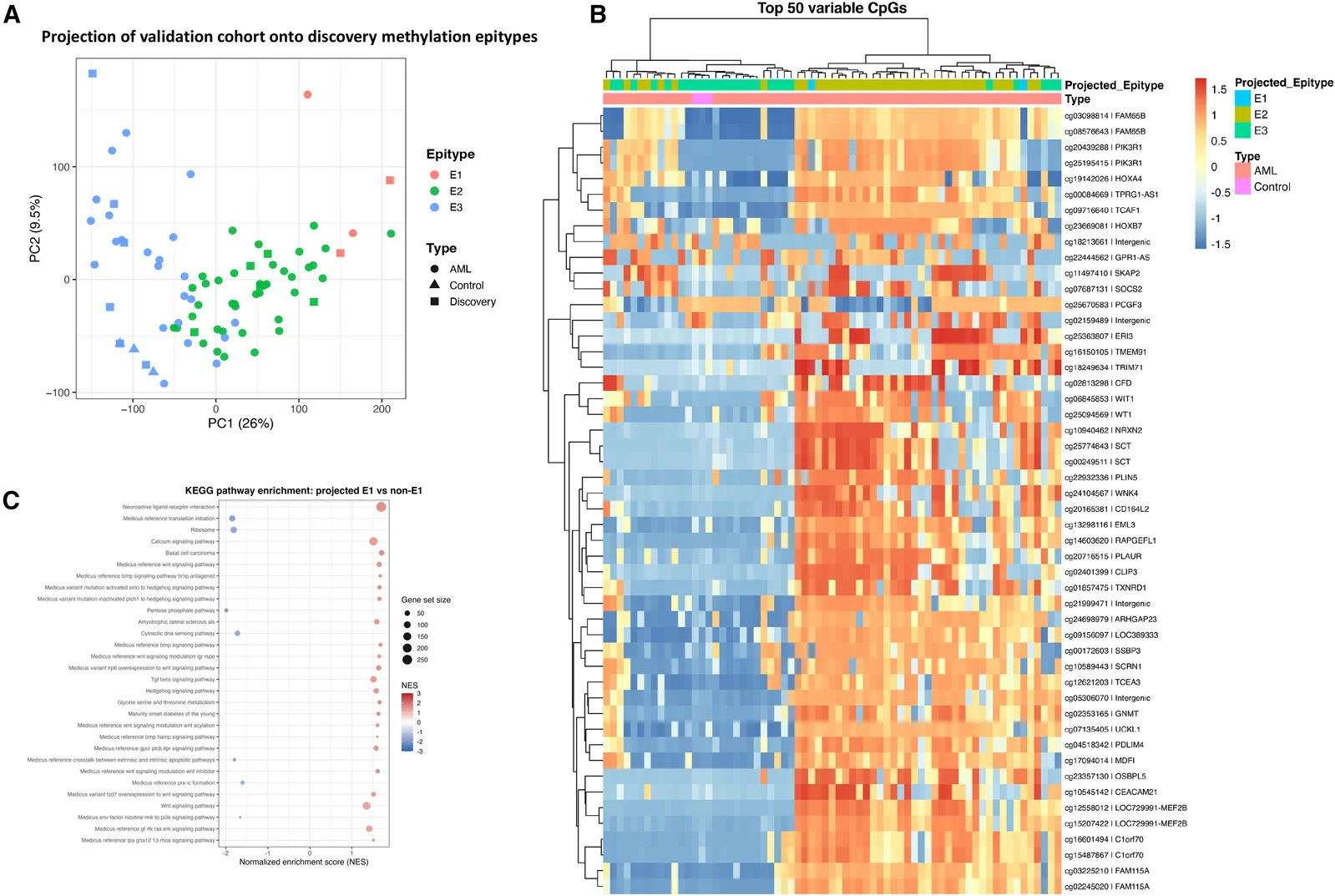
External validation of methylation-defined epitypes in pediatric AML (A) PCA projection of validation samples onto discovery epitype space, colored by epitype (E1-E3) and shaped by sample type. (B) Heatmap of top 50 variable CpGs showing preserved epitype-specific methylation patterns (scaled β-values). Rows indicate CpGs (with gene annotation); columns represent samples. (C) KEGG pathway enrichment comparing projected E1 versus non-E1 AML samples. Positive NES indicates enrichment in E1; negative NES indicates enrichment in non-E1. Bubble size reflects gene set size.

At the locus level, projected samples retained distinct methylation signatures across highly variable CpGs, including loci mapping to genes such as *PIK3R1*, *HOXA4*, *HOXB7*, and *WT1*, supporting preservation of biologically relevant epigenetic structure. Gene-level aggregation further supported consistent but more diffused methylation heterogeneity across samples. Functionally, pathway enrichment analysis revealed a clear divergence between projected epitypes (Figure 10B). E1-like samples were enriched for developmental and morphogenetic programs, including organ development, epithelial morphogenesis, and neuronal differentiation, along with key signaling pathways such as Wnt, Hedgehog, and TGF-β/BMP signaling. Conversely, non-E1 samples were enriched for proliferative, metabolic, and immune-associated processes, including DNA replication, nucleotide biosynthesis, pentose phosphate pathway activity, and interferon signaling (Figure 10C).

Collectively, these findings indicate that methylation-defined epitypes capture conserved biological programs that remain reproducible in an independent cohort. Although cohort heterogeneity influences sample distribution and epitype representation, the underlying epigenetic and pathway-level distinctions remain robust, supporting the generalizability of the epitype framework across pediatric AML.

## Discussion

This study represents integrative multi-omic analyses of methylation-defined epitypes in IR pediatric AML, a clinically ambiguous subgroup that remains poorly defined by current genomic frameworks. By integrating DNA methylation, transcriptomic, and mutational data, we provide a unified view of disease biology. Importantly, our findings demonstrate that disease heterogeneity is not solely explained by individual genetic alterations, but is better understood at the level of convergent pathway dysregulation. Despite diverse mutational landscapes, distinct epitypes converge on shared biological programs, including immune activation, proliferative signaling, and developmental regulation. This pathway-centric framework highlights how multiple molecular alterations can lead to similar functional outcomes, thereby offering a more robust and biologically meaningful basis for disease classification beyond conventional risk stratification.

We identify three biologically distinct methylation-defined epitypes that reflect underlying functional heterogeneity beyond conventional genomic classification. These findings are consistent with prior work, including the cohort represented by GEO: GSE133986, demonstrating that DNA methylation profiles define biologically and clinically relevant AML subtypes independent of genetic alterations.^20^ Similarly, large-scale epitype analyses have shown that methylation-defined subgroups reflect underlying developmental states and are associated with clinical outcomes.^21^ However, these prior analyses did not focus on IR disease and did not incorporate integrative multi-omic characterization. In contrast, our study specifically interrogates IR-AML and demonstrates that methylation-defined epitypes correspond to distinct biological programs, including stress-response and inflammatory signaling (E1), proliferative and survival pathways (E2), and transcriptional and developmental regulation associated with global hypomethylation (E3).

A key observation in our study is the convergence of heterogeneous mutational profiles onto shared pathway-level programs. Despite substantial variability in individual mutations, pathway enrichment analyses consistently identified dysregulation of core oncogenic signaling networks, including MAPK, PI3K-AKT, JAK-STAT, and Wnt signaling. These results support a framework in which heterogeneous genetic alterations converge on common functional pathways, underscoring the value of pathway-centered models for elucidating AML biology.^12^^,^^22^ By integrating methylation and transcriptomic profiles, we further showed that immune activation represents a shared feature of IR-AML, corroborated across molecular layers, with epitype-specific variation in effect size that is most pronounced in E1.

Gene-level integration revealed varied relationships between DNA methylation and gene expression. Although many genes displayed inverse correlations compatible with canonical epigenetic repression, we also observed both concordant and discordant patterns, pointing to context-dependent regulatory mechanisms. This finding aligns with earlier work indicating that methylation influences extend beyond promoter regions and incorporate distal regulatory elements.^23^ From a clinical standpoint, methylation-defined epitypes were linked to distinct survival outcomes, with E1 showing worse survival than E2 and E3. Despite limitations from sample size and wide confidence intervals, the reproducible separation of survival curves across both EFS and OS implies that these epitypes reflect clinically meaningful heterogeneity within IR-AML. The enrichment of immune, inflammatory, and stress-response pathways in E1, including TP53-associated signaling, further suggests that this subgroup may represent a biologically aggressive disease state that is not fully captured by conventional risk stratification.^20^ Future assessment of this epitype in larger, independent cohorts will be critical for establishing its prognostic relevance and its potential role in improving risk classification.

Projection-based mapping to the independent pediatric AML methylation cohort (GEO: GSE133986) further demonstrated that epitype-associated methylation programs are only partially conserved across wider AML populations. While full concordance was not anticipated due to differences in cohort composition, projected samples occupied a comparable methylation space and retained consistent pathway-level signatures. Collectively, these results suggest that methylation-defined epitypes represent conserved biological states while simultaneously capturing context-dependent variation across heterogeneous AML cohorts.

A key strength of this study is its integrated biological approach to IR pediatric AML, rather than focusing on individual markers. This matters because IR-AML constitutes a clinically heterogeneous category, and classification based on conventional cytogenetics or mutations may not adequately account for differences in disease behavior. By combining DNA methylation, mutational, transcriptomic, and survival data, the study delivers a more comprehensive view of the molecular programs that shape this subgroup. The identification of methylation-defined epitypes, supported by internal stability assessment and projection-based analysis in an independent pediatric AML cohort, adds biological relevance to the findings. Although the study is exploratory, it provides a strong foundation for future larger studies evaluating epigenetic stratification as an additional tool for refining risk assessment in pediatric AML. However, several limitations warrant acknowledgment. The relatively small sample size limits statistical power, particularly for survival analyses and mutation frequency estimates, and contributes to wide confidence intervals. Including variants of uncertain significance using relaxed filtering criteria may influence mutation frequency estimates; however, this approach enabled comprehensive pathway-level characterization. In addition, the external validation cohort included a broader range of pediatric AML subtypes rather than exclusively IR cases, and therefore supports biological reproducibility rather than risk-specific generalizability. Finally, we did not apply batch correction between datasets in which batch and biological group were confounded, to avoid removing the true biological signal.

In conclusion, our findings support a model in which pathway-level convergence, rather than individual genetic events, underlies the biological and clinical heterogeneity of IR pediatric AML. This framework underscores that distinct molecular alterations can converge on common functional pathways, providing a biologically meaningful basis for disease classification and highlighting potential avenues for pathway-directed therapeutic strategies.

### Limitations of the study

The study has several limitations to consider when interpreting the findings. As an exploratory investigation of a rare and clinically heterogeneous subgroup, our cohort size constrained statistical power for survival analyses and mutation frequency estimates; therefore, prognostic relationships, including the worse outcomes seen in E1, are presented as hypothesis-generating, and their agreement across both event-free and OS indicates the need for further assessment. To support comprehensive pathway-level characterization, variants of uncertain significance were kept using less stringent filtering criteria, which may affect mutation frequency estimates while leaving the pathway-level convergence that is the main finding of this work unchanged. The independent cohort (GEO: GSE133986) included a wider range of pediatric AML cases and contained methylation data only; its consistency with our epitype-associated programs thus supports biological reproducibility, although risk-specific and multi-omic validation still needs to be demonstrated. Validation in larger, multicenter IR cohorts will further determine the clinical value of methylation-defined epitypes for improving risk stratification in pediatric AML.

## Resource availability

### Lead contact

Requests for further information and resources should be directed to and will be fulfilled by the lead contact, Jagdish Prasad Meena (drjpmeena@yahoo.com).

### Materials availability

This study did not generate new unique reagents.

### Data and code availability

• The DNA methylation array, whole-exome sequencing, and RNA-seq data derived from pediatric patient samples reported in this study cannot be deposited in a public repository because of patient privacy and consent restrictions and the conditions of the Institutional Ethics Committee approval (IEC-383/02.07.2021, RP-12/2021). To request access, contact the lead contact, Jagdish Prasad Meena (drjpmeena@yahoo.com); de-identified data will be shared upon reasonable request and approval of a data use agreement by the Institutional Ethics Committee, All India Institute of Medical Sciences, New Delhi.
• All other data, including de-identified clinical and survival data reported in this paper, will be shared by the lead contact upon request.
• This paper analyzes existing, publicly available data, accessible at GEO: GSE133986.
• This paper does not report original code.
• Any additional information required to reanalyze the data reported in this paper is available from the lead contact upon request.

## Novelty and impact

This study presents an integrative multi-omic analysis of intermediate-risk pediatric acute myeloid leukemia, a subgroup that remains poorly defined by current genomic approaches. Our results show that methylation-defined epitypes capture biologically distinct states, while pathway-level convergence explains shared disease behavior across genetically diverse cases. This work supports the use of pathway-based models for disease classification.

## Acknowledgments

The authors acknowledge the support of the All India Institute of Medical Sciences, New Delhi, for providing funding (Grant No- F.8-AC-23/2020/RS) and infrastructural facilities for this study. We sincerely thank the patients and their families for their participation and consent. We also acknowledge the Pediatric Oncology team, Department of Pediatrics, 10.13039/501100007338AIIMS, New Delhi, for their valuable clinical support and assistance throughout the study. Funding: This study was supported by a grant from the All India Institute of Medical Sciences (AIIMS), New Delhi, India (grant no. F.8-AC-23/2020/RS).

## Author contributions

J.P.M. and R.A.M. conceived the study, designed the analytical framework, supervised the project, secured funding, oversaw project administration, interpreted the findings, and critically revised the manuscript. H.M. performed the patient recruitment, sample processing, whole-exome sequencing, DNA methylation, transcriptomic, and integrative multi-omic analyses; curated and analyzed the data; generated the figures; interpreted the results; and wrote the manuscript. A.K.G., S.B., A.C., P.T., A.S., G.K.V., J.D., D.P., and R.S. contributed to patient recruitment, clinical characterization, sample acquisition, laboratory investigations, resources, and critical review of the manuscript. R.Y., L.S., A.K.P., P.K., S. Bh., and R.K.M. assisted with formal analysis and reviewed the manuscript.

H.G. contributed to formal analysis, manuscript preparation, and critical revision. J.P.M. and R.A.M. provided overall supervision of the study, contributed to study design and interpretation of the findings, and critically revised the manuscript for important intellectual content. All authors reviewed, edited, and approved the final version of the manuscript and agree to be accountable for all aspects of the work.

## Declaration of interests

The authors declare no competing interests.

## STAR★Methods

### Key resources table

**Table:** 

REAGENT or RESOURCE	SOURCE	IDENTIFIER
**Biological samples**		

Peripheral blood and bone marrow aspirates from pediatric patients with AML	Division of Pediatric Oncology, AIIMS, New Delhi	N/A

**Chemicals, peptides, and recombinant proteins**		

Histopaque-1077	Sigma-Aldrich	Cat# 10771

**Critical commercial assays**		

QIAamp DNA Blood Mini Kit	Qiagen	Cat# 51104
RNA extraction kit	Qiagen	Cat# 74104
EZ DNA Methylation Kit	Zymo Research	Cat# D5001
Twist Library Preparation EF Kit	Twist Bioscience	Cat# 101058
Twist Comprehensive Exome Panel	Twist Bioscience	Cat# 102033
KAPA RNA HyperPrep Kit	Roche	Cat# 8105952001
KAPA RiboErase (HMR)	Roche	Cat# 7962274001
Agencourt AMPure XP beads	Beckman Coulter	Cat# A63882
D1000 ScreenTape	Agilent	Cat# 5067-5582
dsDNA Reagent Kit (1–6,000 bp) for Fragment Analyzer	Agilent	Cat# 5191-6576

**Deposited data**		

Pediatric AML DNA methylation data (Infinium MethylationEPIC), independent validation cohort	Yamato et al.^24^	GEO: GSE133986
Human reference genome GRCh38	Genome Reference Consortium	https://www.ncbi.nlm.nih.gov/grc/human

**Software and algorithms**		

R 4.5.0	R Core Team	https://www.r-project.org/
minfi 1.54.1	Aryee et al.^25^	https://bioconductor.org/packages/minfi
limma 3.64.3	Ritchie et al.^26^	https://bioconductor.org/packages/limma
DMRcate 3.4.1	Peters et al.^27^	https://bioconductor.org/packages/DMRcate
missMethyl 1.42.0	Phipson et al.^28^	https://bioconductor.org/packages/missMethyl
IlluminaHumanMethylationEPICanno.ilm10b4.hg19 0.6.0	Hansen^29^	https://bioconductor.org/packages/IlluminaHumanMethylationEPICanno.ilm10b4.hg19
GenomeStudio v2011.1	Illumina	https://www.illumina.com
clusterProfiler 4.16.0	Yu et al.^30^	https://bioconductor.org/packages/clusterProfiler
ReactomePA 1.52.0	Yu and He^31^	https://bioconductor.org/packages/ReactomePA
FastQC v0.12.1	Andrews^32^	https://www.bioinformatics.babraham.ac.uk/projects/fastqc/
Trimmomatic 0.41	Bolger et al.^33^	https://github.com/usadellab/Trimmomatic
BWA-MEM2 2.3	Vasimuddin et al.^34^	https://github.com/lh3/bwa
Picard 3.5.0	Broad Institute	https://broadinstitute.github.io/picard/
GATK 4.6.2.0	McKenna et al.^35^	https://gatk.broadinstitute.org/
ANNOVAR 2025-03-02	Wang et al.^36^	https://annovar.openbioinformatics.org/
maftools 2.24.0	Mayakonda et al.^37^	https://bioconductor.org/packages/maftools
ComplexHeatmap 2.24.1	Gu et al.^38^	https://bioconductor.org/packages/ComplexHeatmap
STAR 2.7.11b	Dobin et al.^39^	https://github.com/alexdobin/STAR
DESeq2 1.48.2	Love et al.^40^	https://bioconductor.org/packages/DESeq2
fgsea 1.34.2	Korotkevich et al.^41^	https://bioconductor.org/packages/fgsea
msigdbr 25.1.1	Dolgalev^42^	https://cran.r-project.org/package=msigdbr
org.Hs.e.g.,.db 3.21.0	Carlson^43^	https://bioconductor.org/packages/org.Hs.eg.db
apeglm 1.30.0	Zhu et al.^44^	https://bioconductor.org/packages/apeglm
matrixStats 1.5.0	Bengtsson^45^	https://cran.r-project.org/package=matrixStats
pheatmap 1.0.13	Kolde^46^	https://cran.r-project.org/package=pheatmap
circlize 0.4.17	Gu et al.^47^	https://cran.r-project.org/package=circlize
ggplot2 4.0.1	Wickham^48^	https://ggplot2.tidyverse.org
data.table 1.17.8	Barrett et al.^49^	https://cran.r-project.org/package=data.table
EnhancedVolcano 1.26.0	Blighe et al.^50^	https://bioconductor.org/packages/EnhancedVolcano
cluster 2.1.8.1	Maechler et al.^51^	https://cran.r-project.org/package=cluster
survival 3.8.3	Therneau^52^	https://cran.r-project.org/package=survival
survminer 0.5.1	Kassambara et al.^53^	https://cran.r-project.org/package=survminer

### Experimental model and study participant details

#### Ethics approval

Institutional Ethics Committee approval nos. IEC-383/02.07.2021, RP-12/2021.

Ethical approval was obtained from the institutional ethics committee. The approval reference numbers for the primary site, the Institute Ethics Committee of the All India Institute of Medical Sciences, New Delhi, India, is IEC-383/02.07.2021, RP-12/2021. The study was conducted in accordance with the principles of the Declaration of Helsinki, and all data collection and handling procedures complied with the applicable ethical requirements.

#### Patients and ethics approval

The study was approved by the Institutional Ethics Committee of the All India Institute of Medical Sciences (AIIMS), New Delhi (IEC-383/02.07.2021, RP-12/2021). Informed consent was obtained from legal guardians and assent, if applicable, and the study was conducted in accordance with the Declaration of Helsinki. Clinical data and treatment outcomes were recorded in accordance with the approved protocol, and all methods were performed in accordance with the relevant guidelines and regulations.

#### Study cohort

A total of 76 pediatric patients (age <18 years) with AML were screened at the Division of Pediatric Oncology, Department of Pediatrics, AIIMS, New Delhi. Fifteen patients were excluded based on predefined criteria: AML associated with Down syndrome (*n* = 2), acute promyelocytic leukemia (*n* = 3), myeloid sarcoma (*n* = 1), juvenile myelomonocytic leukemia (*n* = 1), T cell acute lymphoblastic leukemia (*n* = 1), chronic myeloid leukemia (*n* = 1), B cell acute lymphoblastic leukemia (*n* = 1), treatment initiated outside the study center (*n* = 2), and insufficient sample for cytogenetic and genomic analysis (*n* = 3). Peripheral blood or bone marrow aspirates were collected from the remaining 61 patients.

Cytogenetic analysis identified 20 patients (32.8%) with a normal karyotype (CN-AML). Of these, 13 cytogenetically normal patients classified as intermediate risk according to European LeukemiaNet (ELN) 2017 criteria and meeting all inclusion criteria, with matched DNA methylation and whole-exome sequencing data, formed the integrative multi-omic cohort. Whole-transcriptome sequencing data were available for 5 patients and 3 controls.

#### Independent validation cohort

Publicly available Infinium MethylationEPIC data from an independent cohort of 64 pediatric patients with *de novo* AML (GEO: GSE133986; Yamato et al.) were used for external validation. Raw IDAT files were downloaded from the Gene Expression Omnibus (GEO).

### Method details

#### Sample processing and DNA extraction

Mononuclear cells were isolated from peripheral blood or bone marrow aspirates by density gradient centrifugation using Histopaque-1077 (Sigma-Aldrich) according to the manufacturer’s instructions. Genomic DNA was extracted from mononuclear cells using the QIAamp DNA Blood Mini Kit (Qiagen) according to the manufacturer’s protocol.

#### Genome-wide DNA methylation profiling

Only samples passing DNA quality control were processed. Genomic DNA (500 ng) was bisulfite-converted using the EZ DNA Methylation Kit (Zymo Research) according to Illumina’s recommendations. Converted DNA was whole-genome amplified, enzymatically fragmented, precipitated, and resuspended in hybridization buffer before loading onto Infinium MethylationEPIC BeadChips (Illumina), which interrogate more than 850,000 CpG sites. Hybridization was performed for 18 h at 48°C, followed by washing, single-base extension, and fluorescent staining. BeadChips were scanned on an Illumina iScan system, and control dashboard quality metrics were assessed using GenomeStudio (v2011.1).

#### DNA methylation data preprocessing

Raw IDAT files were imported into R 4.5.0 using minfi, and background correction and dye-bias normalization were performed using the normal-exponential out-of-band (NOOB) method (preprocessNoob). Detection *p* values were computed for each probe; samples in which ≥5% of probes had detection *p* >0.01 were excluded, and probes with detection *p* <0.01 in fewer than 95% of samples were removed. Probes overlapping SNPs at the CpG site or single-base extension site and probes on the X and Y chromosomes were removed. Probe annotation was based on IlluminaHumanMethylationEPICanno.ilm10b4.hg19. β values were used for clustering and visualization, and M values (log2[β/(1 − β)]) for statistical modeling.

#### Differential methylation analysis

Differential methylation between groups was assessed using linear models with empirical Bayes moderation in limma, with sample group as the covariate. *p* values were adjusted using the Benjamini-Hochberg method, and effect sizes were reported as Δβ (difference in group mean β).

#### Identification of methylation-defined epitypes

Epitypes were derived from IR-AML samples only. The CpG universe was restricted to probes in promoter regions (TSS200, TSS1500, 5′UTR, or first exon) or CpG island-proximal regions (islands, shores, and shelves), and the 30,000 most variable CpGs (by β-value variance) within this universe were selected. β values were centered and scaled across IR-AML samples, and PCA was performed. Principal components cumulatively explaining ≥90% of the variance (maximum of 10) were used for k-means clustering (K = 3; 50 random starts; maximum 1,000 iterations; seed 1234), yielding epitypes E1–E3. Control samples were projected into the same principal-component space using the IR-AML-derived centering, scaling, and rotation.

#### Sensitivity and stability analyses

To assess robustness to feature selection, clustering was repeated using the 50,000 most variable CpGs across the whole array, the 20,000 most variable promoter CpGs, and the 50,000 most variable CpG island-proximal CpGs, and assignments were compared across feature sets. The distribution of selected CpGs across CpG island contexts was compared with the background of all filtered probes. Association between epitype assignments and technical covariates (BeadChip slide and array position) was tested using Fisher’s exact test. Cluster separation and stability were further evaluated across K = 2–6 for promoter-restricted, CpG island-proximal, and whole-array feature sets using silhouette width and bootstrap-based best-match Jaccard similarity.

#### Epitype-associated CpGs

CpGs differing across epitypes were identified within IR-AML samples using a moderated F-test (one-way ANOVA framework) in limma on β values, fitting a cell-means model with epitype as the factor. CpGs were ranked by adjusted *p* value, then by the maximum pairwise difference in mean β between epitypes (Δβmax), then by F-statistic. The top 50 and 100 CpGs were visualized as heatmaps of β values (0–1) with clinical annotations using ComplexHeatmap, with hierarchical clustering of rows and columns (Euclidean distance, complete linkage).

#### Methylation-based gene set enrichment analysis

Gene set enrichment analysis was performed on Funnorm-normalized M values of the 30,000 most variable CpGs across the array. For each contrast (IR-AML versus control, and each epitype versus the remaining IR-AML samples), a pooled-variance two-sample t-statistic was computed per CpG and summarized per gene as the median across associated CpGs; genes were mapped to Entrez identifiers using org.Hs.eg.db. Ranked lists were analyzed with fgsea (5,000 permutations) against MSigDB GO biological process, molecular function, cellular component, and Reactome gene sets retrieved with msigdbr, restricted to sets of 15–1,000 genes. Pathways were ranked by Benjamini-Hochberg-adjusted *p* value and absolute normalized enrichment score (NES).

#### Whole-exome sequencing

Libraries were prepared from 100 ng of genomic DNA using the Twist Library Preparation EF Kit (Twist Bioscience). DNA was enzymatically fragmented to approximately 250 bp, end-repaired, A-tailed, and ligated to adapters. Adapter-ligated products were purified using Agencourt AMPure XP beads (Beckman Coulter) and amplified by PCR (98°C for 45 s; 13 cycles of 98°C for 15 s, 60°C for 30 s, and 72°C for 30 s; final extension at 72°C for 1 min). Exonic regions were captured using the Twist Comprehensive Exome Panel (Twist Bioscience) by hybridization to biotinylated probes, followed by streptavidin bead capture, magnetic pull-down, and post-capture PCR amplification under the same cycling conditions. Fragment size distribution of the final libraries was assessed on a TapeStation using D1000 ScreenTapes (Agilent) or on a Fragment Analyzer using the dsDNA Reagent Kit (Agilent).

#### Exome data processing and variant calling

Raw FASTQ files were assessed using FastQC. Adapters and low-quality bases were removed using Trimmomatic in paired-end mode, and cleaned reads were aligned to the human reference genome (GRCh38) using BWA-MEM. Alignments were coordinate-sorted, and PCR and optical duplicates were marked using Picard. Variants were called using bcftools mpileup and call and annotated using ANNOVAR with RefGene gene models, gnomAD and ExAC population frequencies, dbNSFP functional predictions, and ClinVar. Annotated variants were converted to Mutation Annotation Format (MAF) and analyzed using maftools.

#### Variant filtering

Because matched germline samples were not available, variants were filtered to enrich for putative somatic alterations. Protein-coding, non-synonymous exonic variants with read depth >50× were retained. Variants with a population allele frequency ≥1% in gnomAD (popmax) or ExAC, variants classified as benign or likely benign in ClinVar, and variants in frequently mutated genes (FLAGS) were excluded. Missense variants were retained if predicted to be damaging by at least one of SIFT (≤0.10), PolyPhen-2 HDIV (≥0.50), or CADD (≥15); truncating, splice-site, and in-frame indel variants were retained irrespective of prediction scores. Variants of uncertain significance meeting these criteria were retained. Tumor sample barcodes were remapped to study identifiers, and only the most deleterious variant per sample-gene pair was retained (nonsense > splice site > frameshift deletion > frameshift insertion > nonstop > translation start site > in-frame deletion > in-frame insertion > missense). Variants in canonical AML driver genes with depth ≤50× were reviewed manually in the Integrative Genomics Viewer.

#### Mutational landscape, driver, and pathway analysis

Tumor mutation burden (TMB) was calculated as the number of non-synonymous variants per megabase, assuming a 38 Mb exome capture size and categorized into tertiles. Ti/Tv ratios and SNV/indel ratios were computed, and cohort-level mutation summaries were visualized using oncoplots, rainfall plots, lollipop plots, and Ti/Tv spectra. Candidate driver genes were identified using the OncodriveCLUST algorithm implemented in maftools (oncodrive; minimum of two mutations per gene; z-score method), complemented by a curated panel of AML driver genes. Mutated genes were grouped into curated pathway panels (RTK–RAS, PI3K–AKT, Wnt, epigenetic regulators, spliceosome, cohesin, and transcription factors), and canonical oncogenic signaling pathways were summarized using maftools. Pathway enrichment of mutated genes was assessed using enrichKEGG (clusterProfiler) and enrichPathway (ReactomePA) with *p* <0.05 and q <0.25. Mutation profiles were compared across epitypes using per-epitype oncoplots and epitype-by-pathway mutation counts.

#### Whole-transcriptome sequencing

Total RNA sequencing libraries were prepared from 100 ng of total RNA using the KAPA RNA HyperPrep Kit (Roche). Ribosomal RNA was depleted using KAPA RiboErase (HMR) (Roche), and the depleted RNA was fragmented using divalent cations at 94°C for 6 min. Fragmented RNA was reverse-transcribed into first-strand cDNA, followed by combined second-strand synthesis and A-tailing, in which dUTP was incorporated into the second strand and dAMP was added to the 3′ ends of double-stranded cDNA. Indexed adapters with 3′ dTMP overhangs were ligated, and adapter-ligated products were purified and amplified using high-fidelity, low-bias PCR (98°C for 45 s; 13 cycles of 98°C for 15 s, 60°C for 30 s, and 72°C for 30 s; final extension at 72°C for 1 min); the dUTP-marked strand was not amplified, preserving strand specificity. Libraries were purified, and fragment size distribution was assessed on a Fragment Analyzer using the dsDNA Reagent Kit (1–6,000 bp; Agilent). Libraries were sequenced on an Illumina NovaSeq X Plus platform (2 × 150 bp paired-end reads; approximately 60 million reads per sample), with up to 75% of bases achieving a quality score of Q30 or higher.

#### RNA-seq data processing and differential expression analysis

Gene-level raw counts were restricted to protein-coding genes, and genes with fewer than 10 total counts across samples were removed. Differential expression between patients and controls was assessed using DESeq2 with size factors estimated using the poscounts method and Wald testing; log2 fold changes were shrunk using apeglm. *p* values were adjusted using the Benjamini-Hochberg method, and genes with FDR <0.20 were considered differentially expressed; this relaxed threshold was applied because of the limited sample size. A variance-stabilizing transformation (blind = FALSE) was applied for PCA and heatmaps of the 50 most significant genes, and volcano plots were generated using EnhancedVolcano.

#### Gene set enrichment analysis

Gene set enrichment analysis was performed using fgsea, with genes ranked by the DESeq2 Wald statistic (patients versus controls). Gene symbols were mapped to Entrez identifiers using org.Hs.eg.db, and ranked lists were tested against Gene Ontology biological process and KEGG gene sets from MSigDB, retrieved using msigdbr and restricted to sets of 15–1,000 genes, with [5,000] permutations. *p* values were adjusted using the Benjamini–Hochberg method, and pathways with FDR <[0.05] were considered significantly enriched. Positive and negative normalized enrichment scores (NES) indicate enrichment in patients and controls, respectively.

#### Integrative methylation and expression analysis

For samples with both methylation and RNA-seq data, gene-level methylation was summarized as the mean β value, and expression as log2-transformed normalized counts. Associations between methylation and expression were assessed for each gene using Spearman correlation. Genes were classified as showing inverse, concordant, or discordant methylation–expression relationships and were integrated with mutation status and functional class in alluvial plots and combined expression–methylation heatmaps annotated by disease status and epitype.

#### Preprocessing of the validation cohort

IDAT files from GSE133986 were processed using the pipeline applied to the discovery cohort, including NOOB normalization, detection p value-based probe filtering, and removal of probes overlapping SNPs, cross-reactive probes, and sex chromosome probes. Only CpGs common to both the discovery and validation cohorts were retained.

#### Unsupervised clustering of the validation cohort

To evaluate whether the validation cohort independently recapitulated structured methylation heterogeneity, unsupervised clustering was performed on highly variable CpGs, and the optimal number of clusters was assessed using silhouette width and bootstrap-based Jaccard similarity across K = 2–6.

#### Projection-based epitype assignment

Epitype centroids (E1–E3) were derived from the discovery cohort as the mean β values of epitype-defining CpGs. Each validation sample was assigned to the nearest epitype based on Pearson correlation with these centroids, and assignment confidence was quantified as the difference between the highest and second-highest correlation coefficients. PCA was performed on the combined discovery and validation datasets to visualize the position of projected samples within the global methylation space. Pathway concordance was assessed by fgsea of KEGG gene sets comparing projected E1 with non-E1 samples.

### Quantification and statistical analysis

All analyses were performed in R 4.5.0. Differential methylation was assessed using moderated t- and F-statistics in limma, and differential expression using the Wald test in DESeq2; *p* values were adjusted for multiple testing using the Benjamini-Hochberg method. Associations between epitypes and technical covariates were tested using Fisher’s exact test.

Overall survival (OS) was defined as the time from diagnosis to death from any cause, and event-free survival (EFS) as the time from diagnosis to relapse, progression, or death times were expressed in months, and patients without an event were censored at last follow-up. Survival was estimated using the Kaplan-Meier method and compared across epitypes using the log-rank test with the survival package. Pairwise hazard ratios with 95% confidence intervals were estimated using Cox proportional hazards regression.

Unless otherwise stated, two-sided *p* <0.05 was considered statistically significant. The number of samples (n) and the statistical tests used for each analysis are reported in the figure legends.

## Supplemental information

Document S1. Figures S1–S6 and Supplemental File 1 Validation of Epitypes
