# Supplementary material for: Integrated multi-omics reveals methylation-linked biological heterogeneity in intermediate-risk pediatric acute myeloid leukemia
Source: iScience. 2026 Nov 2;29(11):118028. doi: 10.1016/j.isci.2026.118028 (PMC13755973; doi:10.1016/j.isci.2026.118028)
Supplement: Supplementary file 1 [file mmc1.pdf]

iScience, Volume *29*

Supplemental information

Integrated multi-omics reveals methylation-linked biological heterogeneity in intermediate-risk pediatric acute myeloid leukemia

Harshita Makkar, Jagdish Prasad Meena, Riyaz Ahmad Mir, Aditya Kumar Gupta, Sameer Bakhshi, Anita Chopra, Pranay Tanwar, Rakhee Yadav, Lata Singh, Harsh Goel, Avanish Kumar Pandey, Pankaj Keshari, Sujata Bhattacharya, Ravi Kumar Majhi, Amitabh Singh, Shreshtha Kaushik, Sanjana Sarangarajan, Ganesh Kumar Viswanathan, Jasmita Dass, Deepam Pushpam, and Rachna Seth

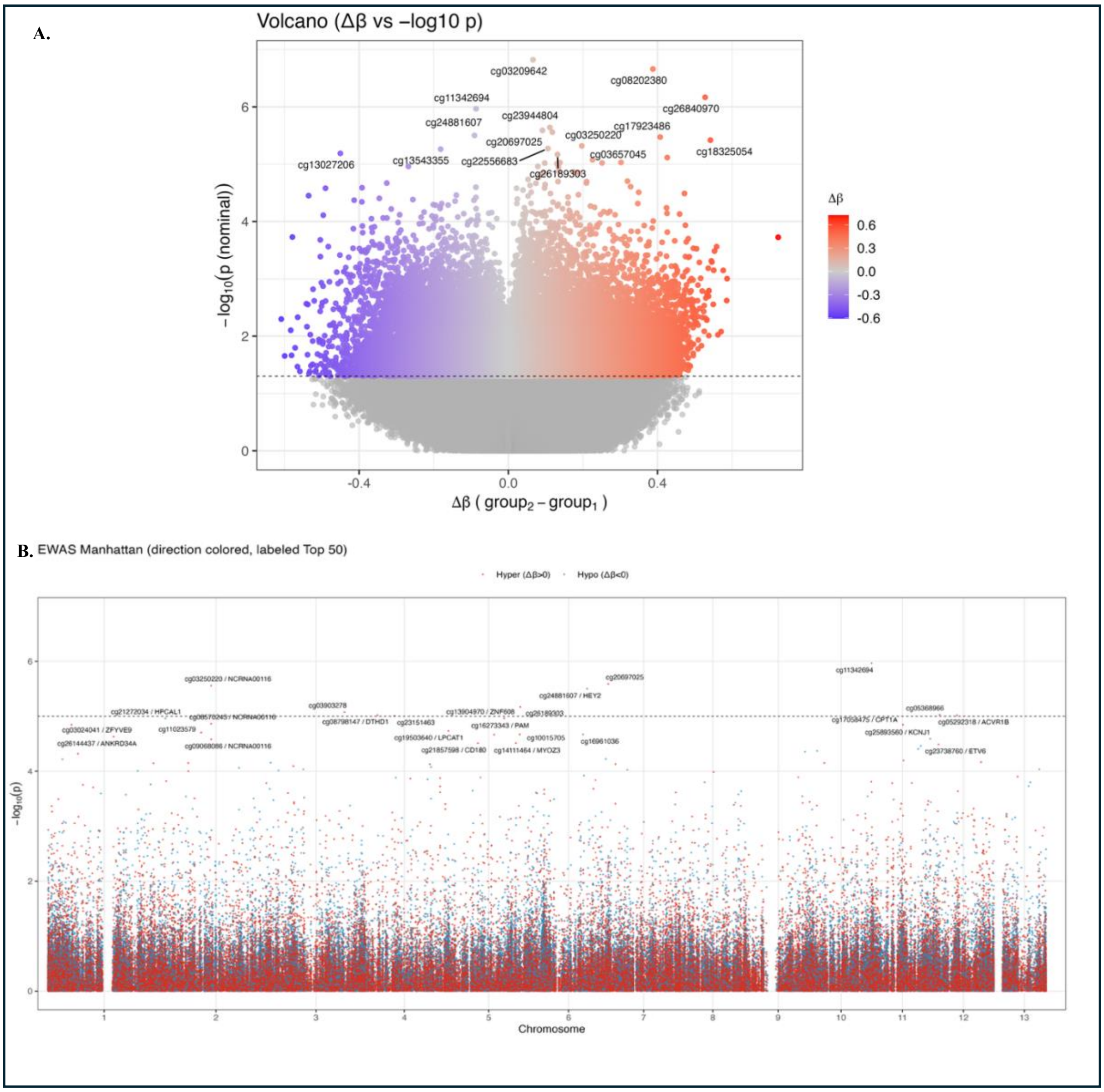

Supplementary Figure 1: A. Volcano plot having Δβ versus −log10(p), top CpGs labeled. The plot shows differential methylation between groups (Δβ vs −log10 p). Significant CpGs ($p < 0.05$) are highlighted and colored by direction (red, hypermethylated; blue, hypomethylated). **B.** Manhattan plot of genome-wide differential methylation (−log10 p) across chromosomal positions. CpGs are colored by direction of methylation change, with top-ranked probes labeled.

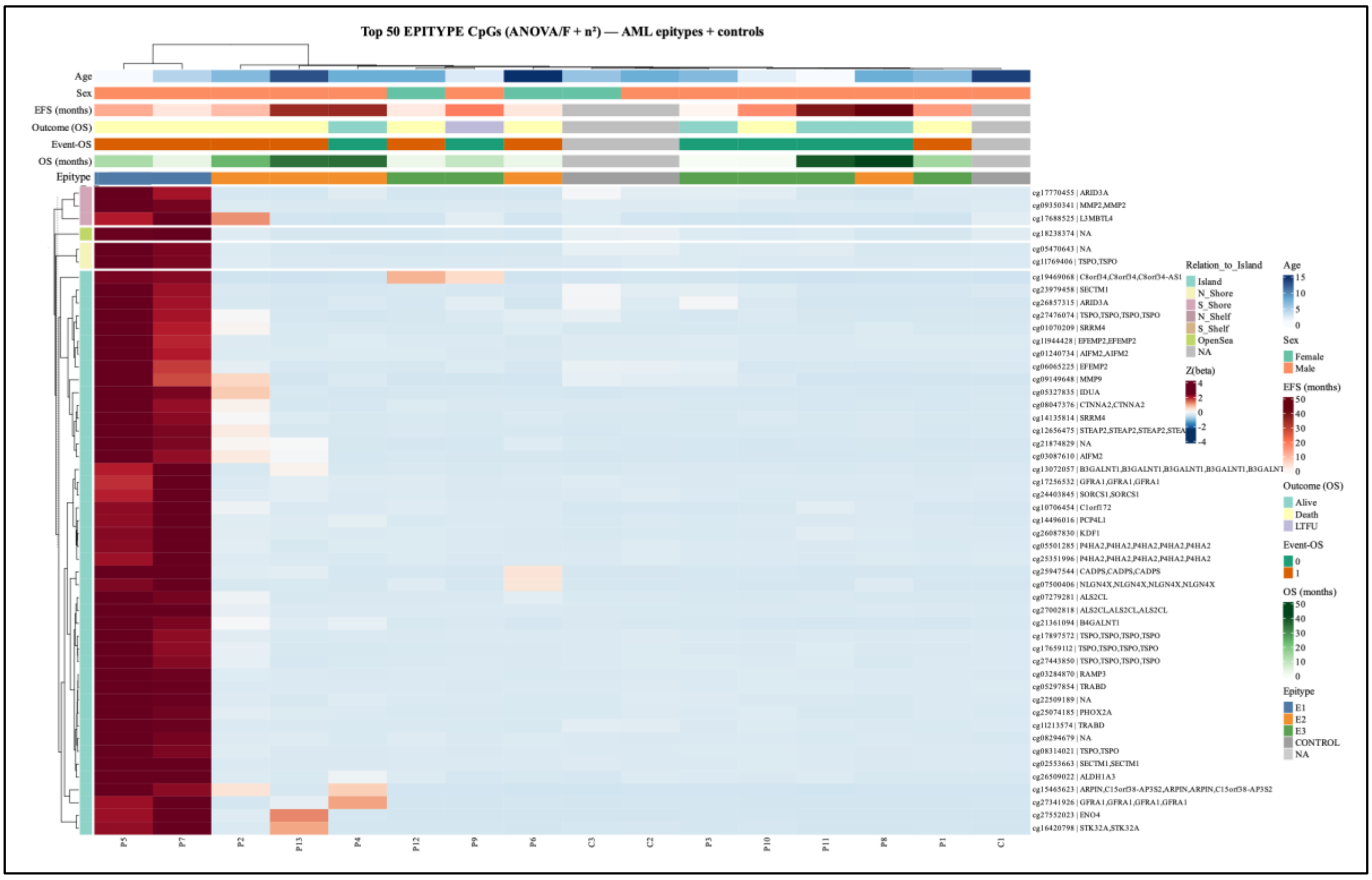

Supplementary Figure 2. Epitype-defining DNA methylation landscape of intermediate-risk AML. Heatmap depicting the top 50 epitype-discriminatory CpG loci in intermediate-risk AML (IR-AML), identified using an overall ANOVA (limma F-test) across methylation epitypes (E1–E3) and ranked by a composite η² score $(-\log10\text{adjustedPvalue})^2 \times |F|$ .Rows represent CpG sites labeled as CpG ID followed by nearest annotated gene(s) and are stratified by CpG island context, ordered with CpG islands first, followed by shores, shelves, open sea, and unannotated regions. Columns correspond to patient samples and controls, annotated with Age, Sex, Event-Free Survival (EFS), Overall Survival (OS), Event-OS status, and Epitype assignment. Heatmap colors represent row-scaled methylation values (Z-scores) (blue, relative hypomethylation; red, relative hypermethylation). Controls cluster distinctly from IR-AML epitypes, highlighting disease-specific epigenetic programs.

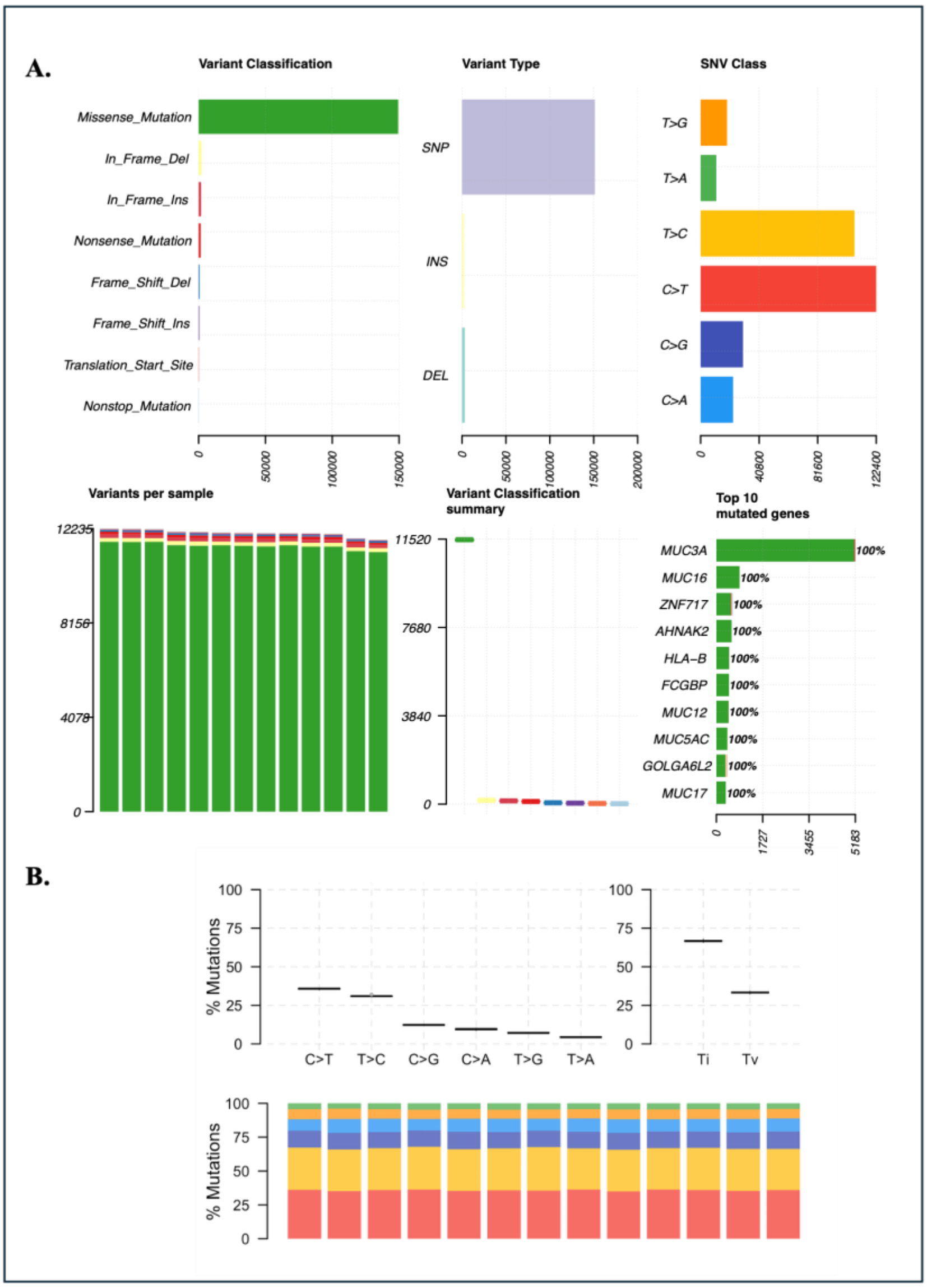

Supplementary Figure 3. Somatic mutation landscape in intermediate-risk AML. (A) Oncoplot of recurrent AML driver gene mutations across IR-AML samples. Columns represent patients and rows represent genes; variant classifications are color-coded. The top barplot shows mutation counts per sample, and the right barplot shows gene-wise mutation frequency. (B) Transition/transversion (Ti/Tv) profile showing the distribution of nucleotide substitution classes across all somatic variants.

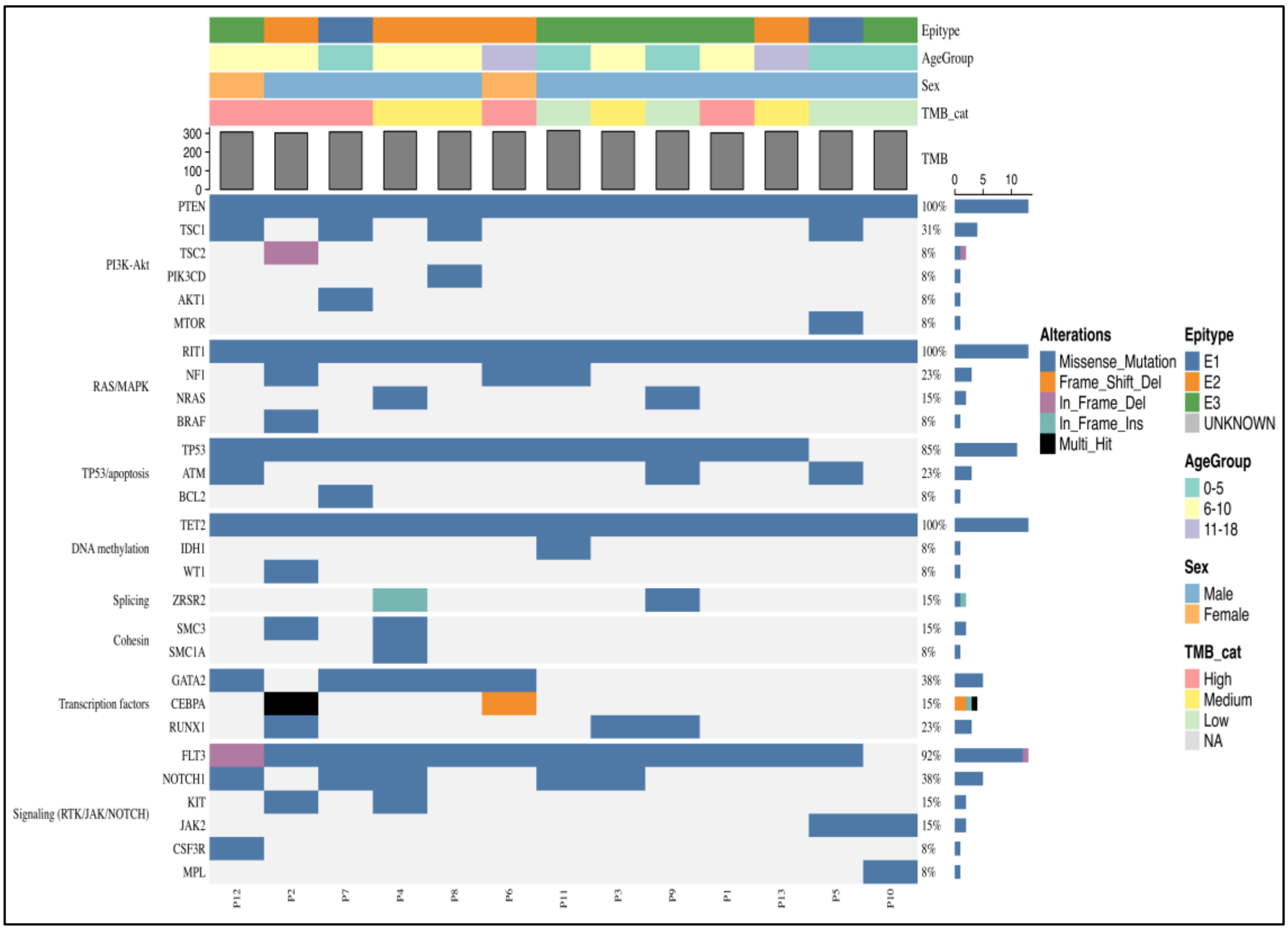

Supplementary Figure 4- Oncoprint of somatic mutations in intermediate-risk AML stratified by methylation epitypes and clinical features. The oncoprint depicts somatic alterations in recurrent AML driver genes grouped by functional pathways, including PI3K–AKT, RAS/MAPK, TP53/apoptosis, DNA methylation, RNA splicing, cohesin complex, transcription factors, and signaling pathways (RTK/JAK/NOTCH). Columns represent individual IR-AML patients, and rows represent genes. Mutation types are color-coded by variant classification, with multi-hit events indicated separately. Top annotation tracks show methylation epitype assignment, age group, sex, tumor mutational burden (TMB) category, and per-sample TMB. Bar plots on the right indicate gene-wise mutation frequencies across the cohort. This figure highlights inter-patient heterogeneity, pathway-level convergence, and the distribution of driver mutations across methylation-defined epitypes.

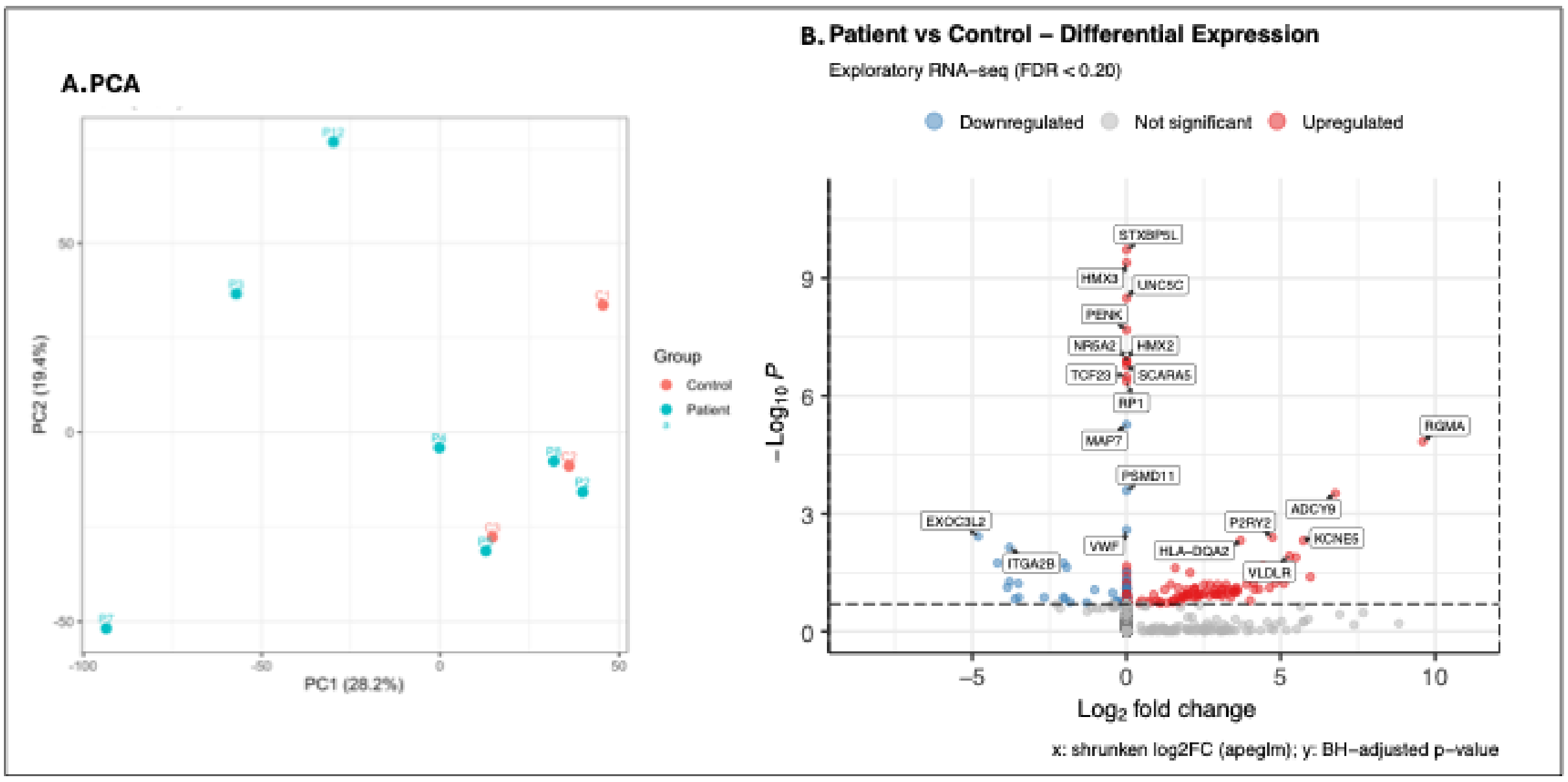

Supplementary Figure 5. Transcriptomic differences between pediatric AML and controls. (A) Principal component analysis (PCA) of variance-stabilized RNA-seq data showing separation between AML and control samples (PC1, 28.2%; PC2, 19.4%). (B) Volcano plot of differential gene expression (FDR < 0.20), with upregulated genes in red and downregulated genes in blue; selected genes are labeled.

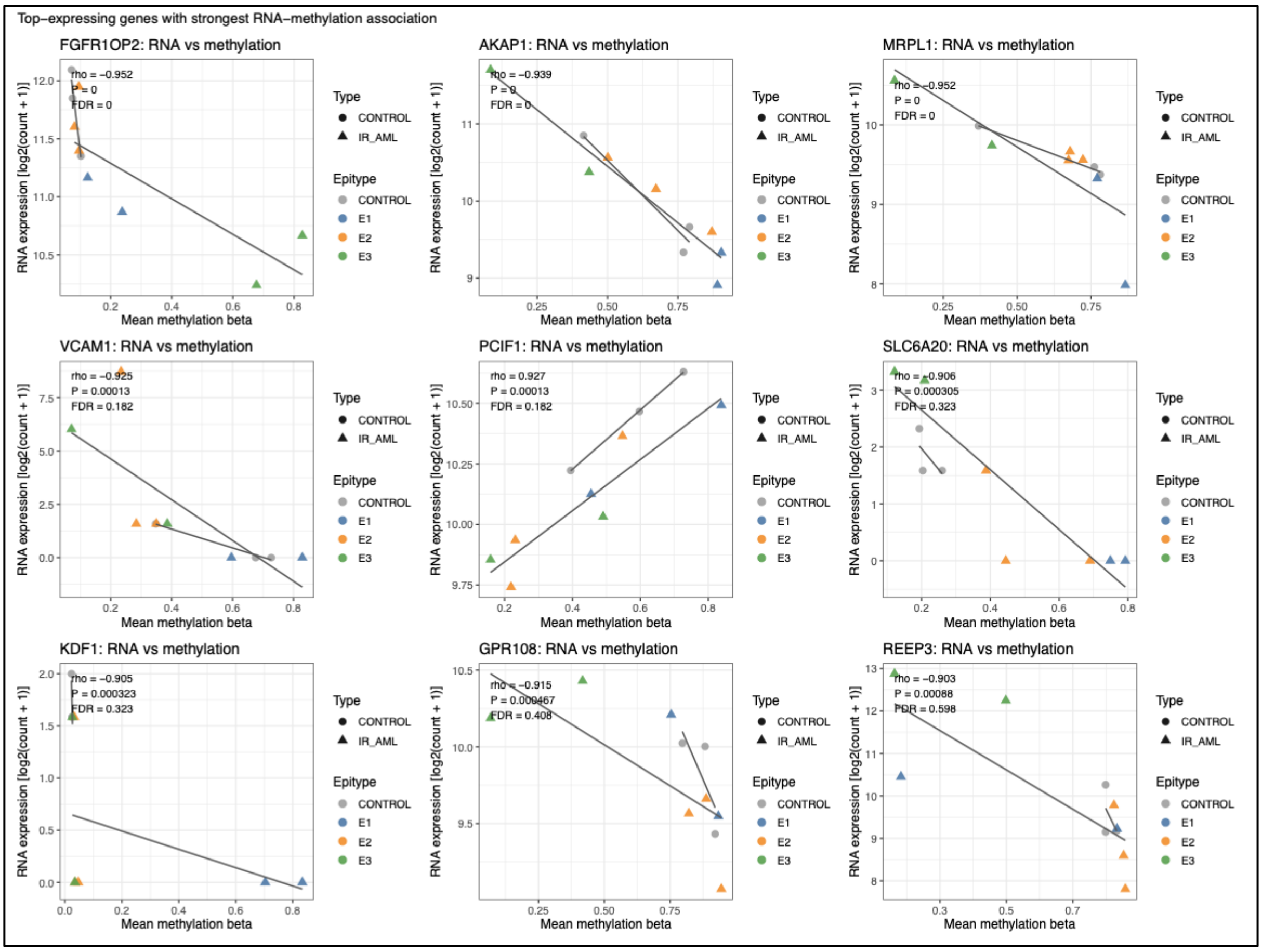

Supplementary Figure 6. RNA–methylation correlation for selected genes in pediatric AML. Scatter plots showing the relationship between mean DNA methylation (β-values) and gene expression (log2-transformed counts) for selected genes with strong RNA–methylation associations. Each point represents a sample, colored by methylation epitype and annotated by disease status (control vs IR-AML). Spearman correlation coefficients (ρ), P values, and FDR-adjusted values are indicated for each gene. Most genes exhibit inverse correlations consistent with epigenetic regulation, while select genes show positive associations, indicating context-dependent regulatory relationships.

Supplementary File 1- Validation of Epitypes

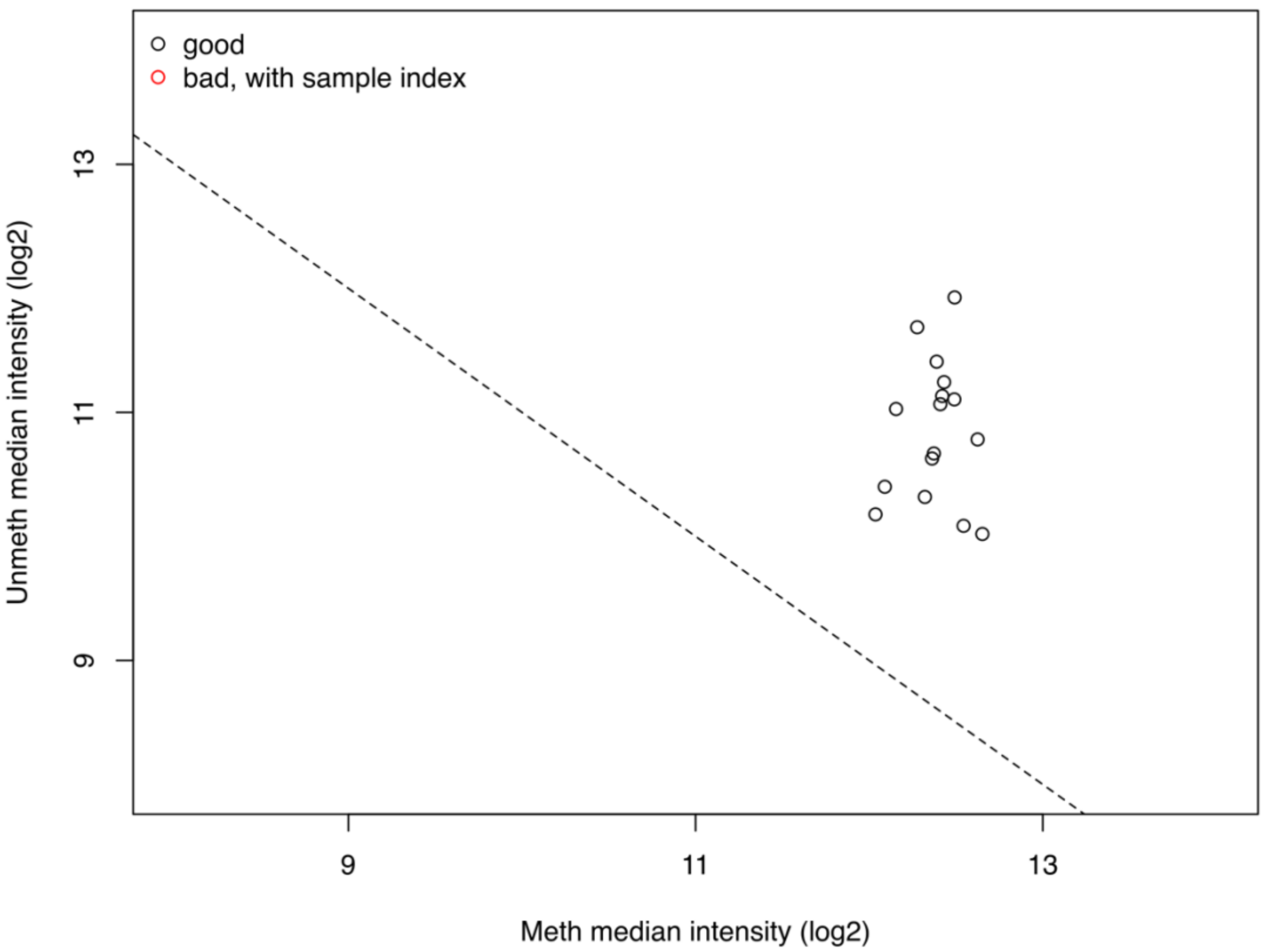

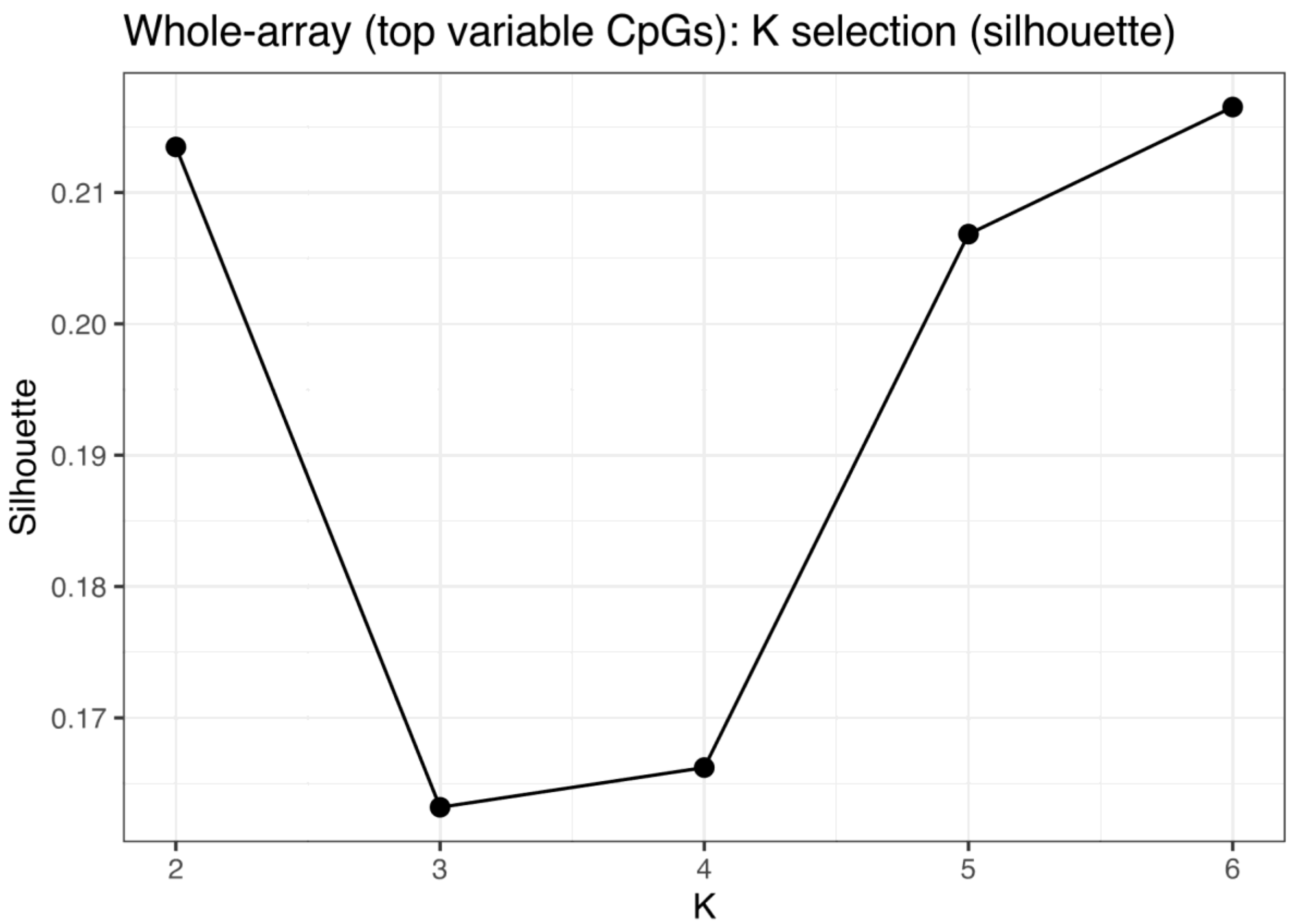
Whole-array (top variable CpGs): K selection (silhouette)
Silhouette
0.21
0.20
0.19
0.18
0.17
2
3
4
5
6
K

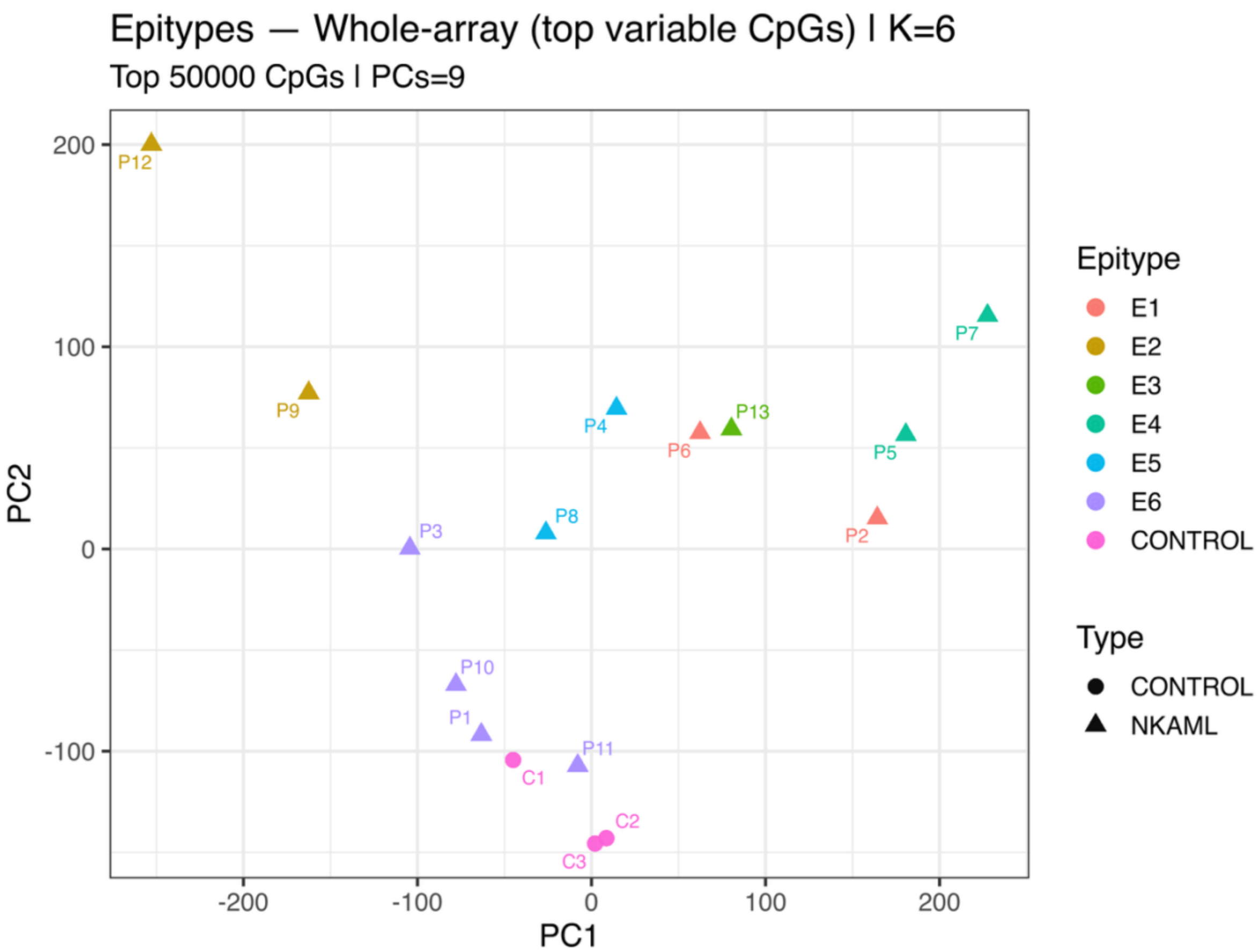
Epitypes — Whole-array (top variable CpGs) | K=6
Top 50000 CpGs | PCs=9
PC2
200
100
0
-100
-200
-100
0
100
200
PC1
P12
P9
P7
P4
P13
P6
P5
P3
P8
P2
P10
P1
P11
C1
C2
C3
Epitype
E1
E2
E3
E4
E5
E6
CONTROL
Type
CONTROL
NKAML

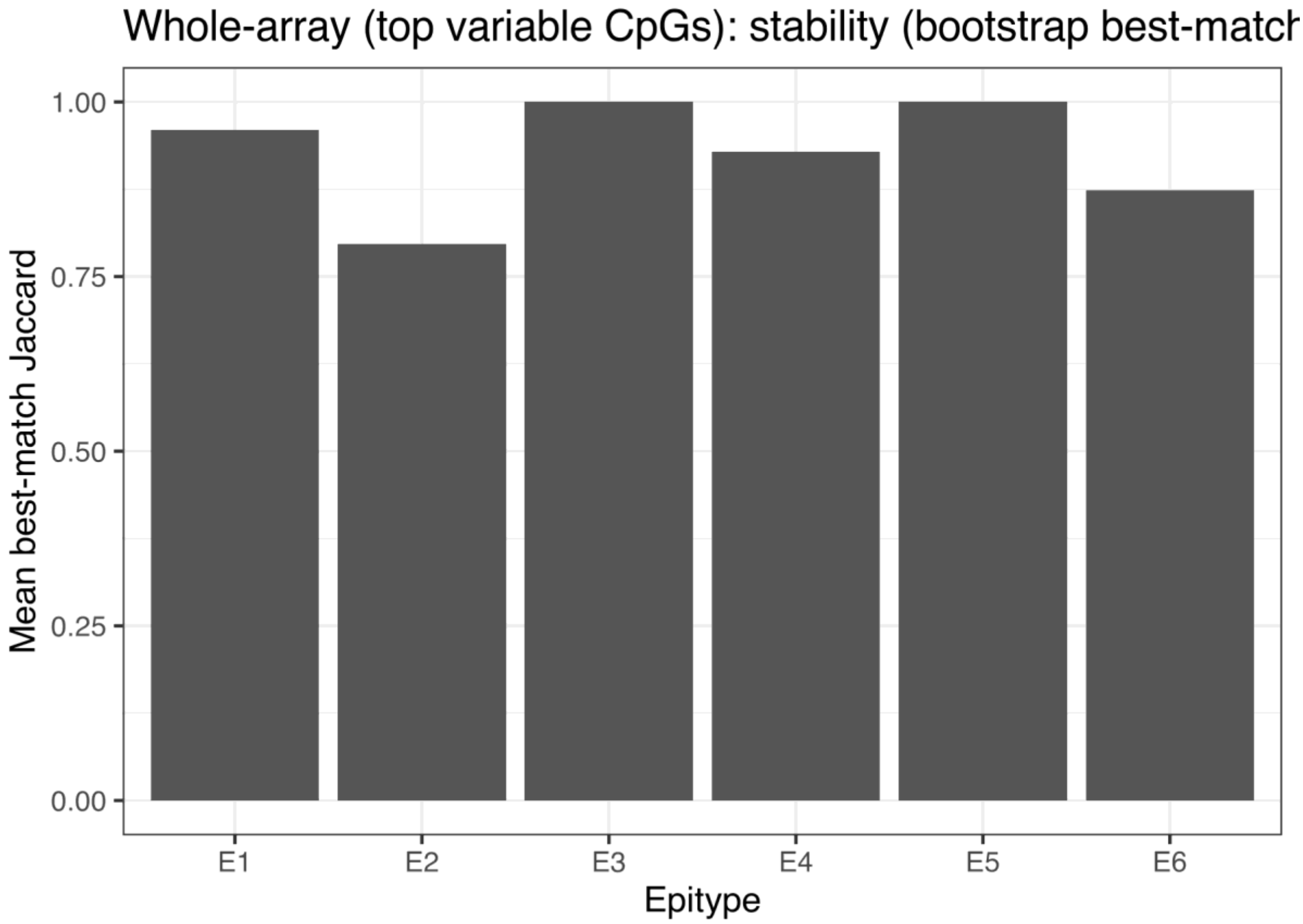

Whole-array (top variable CpGs): stability (bootstrap best-match
Mean best-match Jaccard
1.00
0.75
0.50
0.25
0.00
E1
E2
E3
E4
E5
E6
Epitype

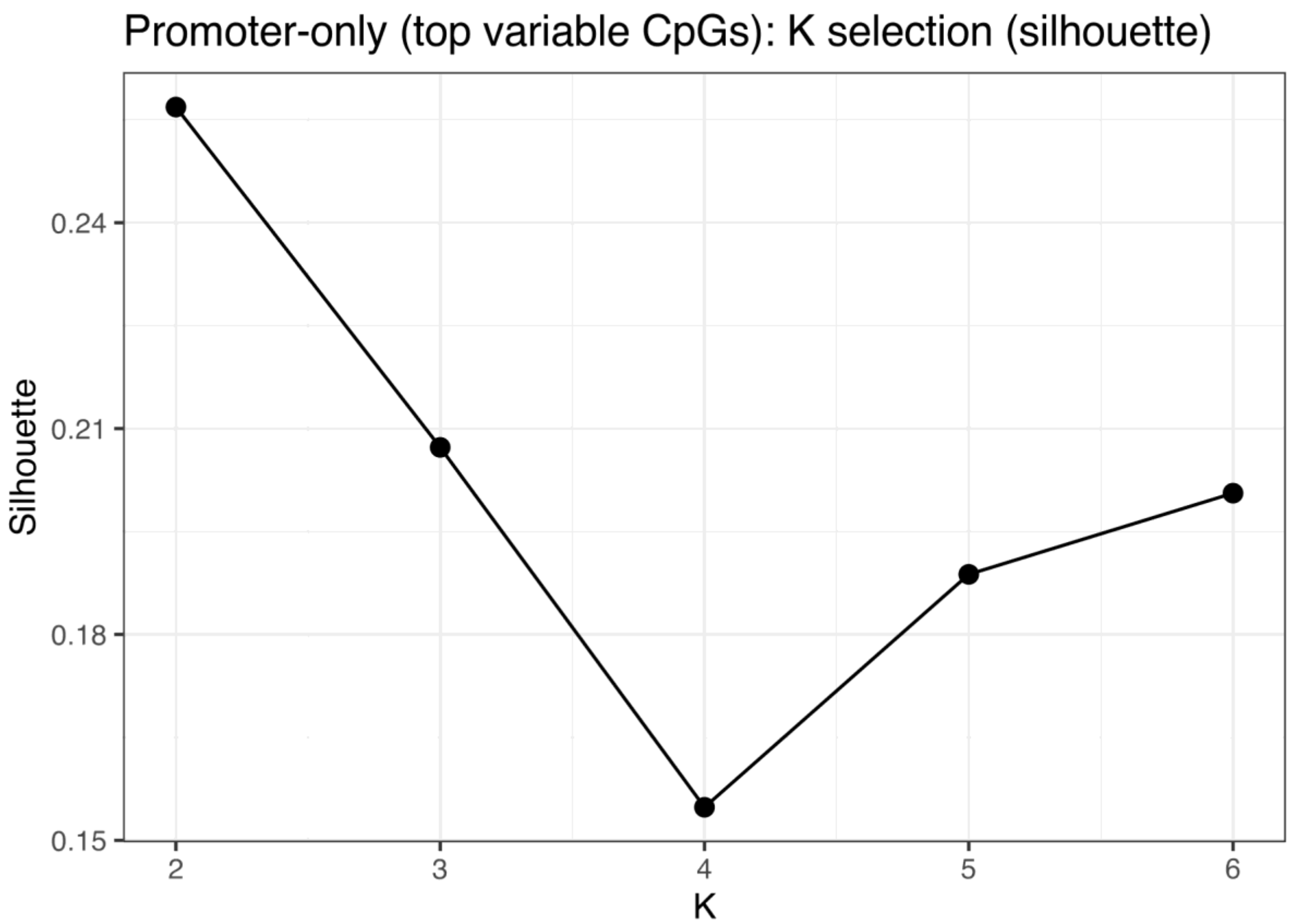
Promoter-only (top variable CpGs): K selection (silhouette)
Silhouette
0.24
0.21
0.18
0.15
2
3
4
5
6
K

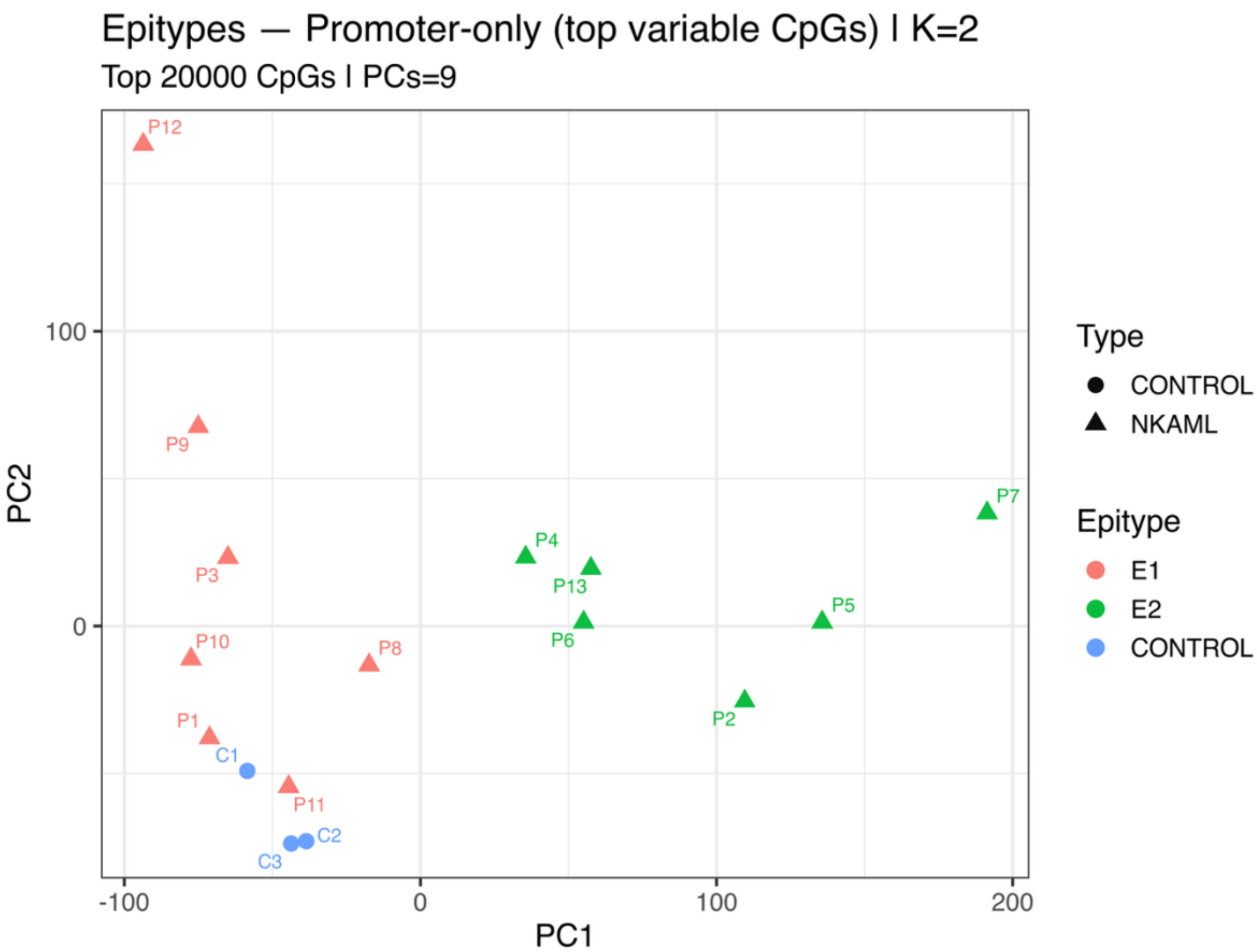
Epitypes — Promoter-only (top variable CpGs) | K=2
Top 20000 CpGs | PCs=9
PC2
100
0
-100
0
100
200
PC1
P12
P9
P3
P10
P1
C1
P11
C2
C3
P8
P4
P13
P6
P2
P5
P7
Type
CONTROL
NKAML
Epitype
E1
E2
CONTROL

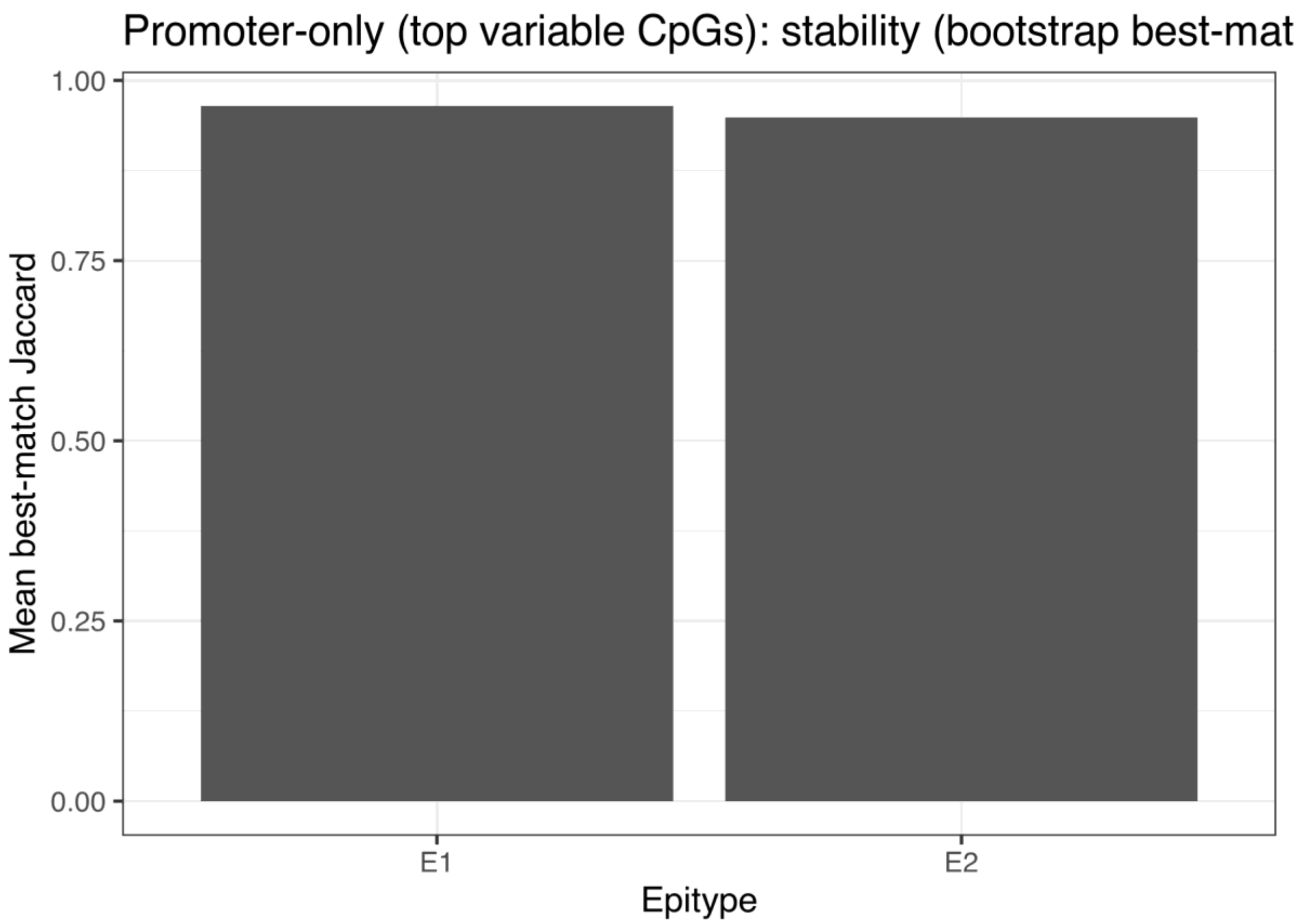
Promoter-only (top variable CpGs): stability (bootstrap best-mat
Mean best-match Jaccard
1.00
0.75
0.50
0.25
0.00
E1
E2
Epitype

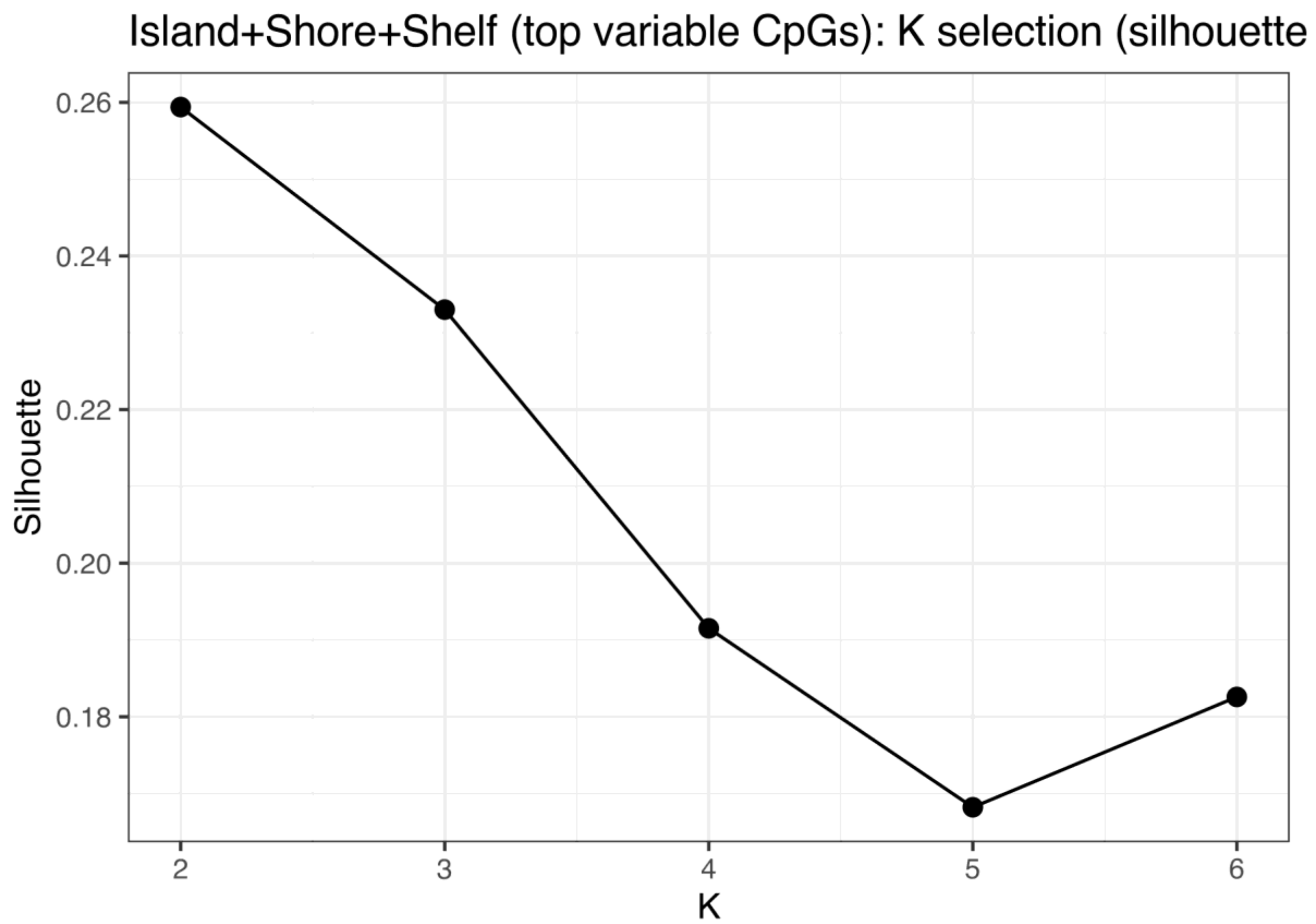
Island+Shore+Shelf (top variable CpGs): K selection (silhouette
Silhouette
0.26
0.24
0.22
0.20
0.18
K
2
3
4
5
6

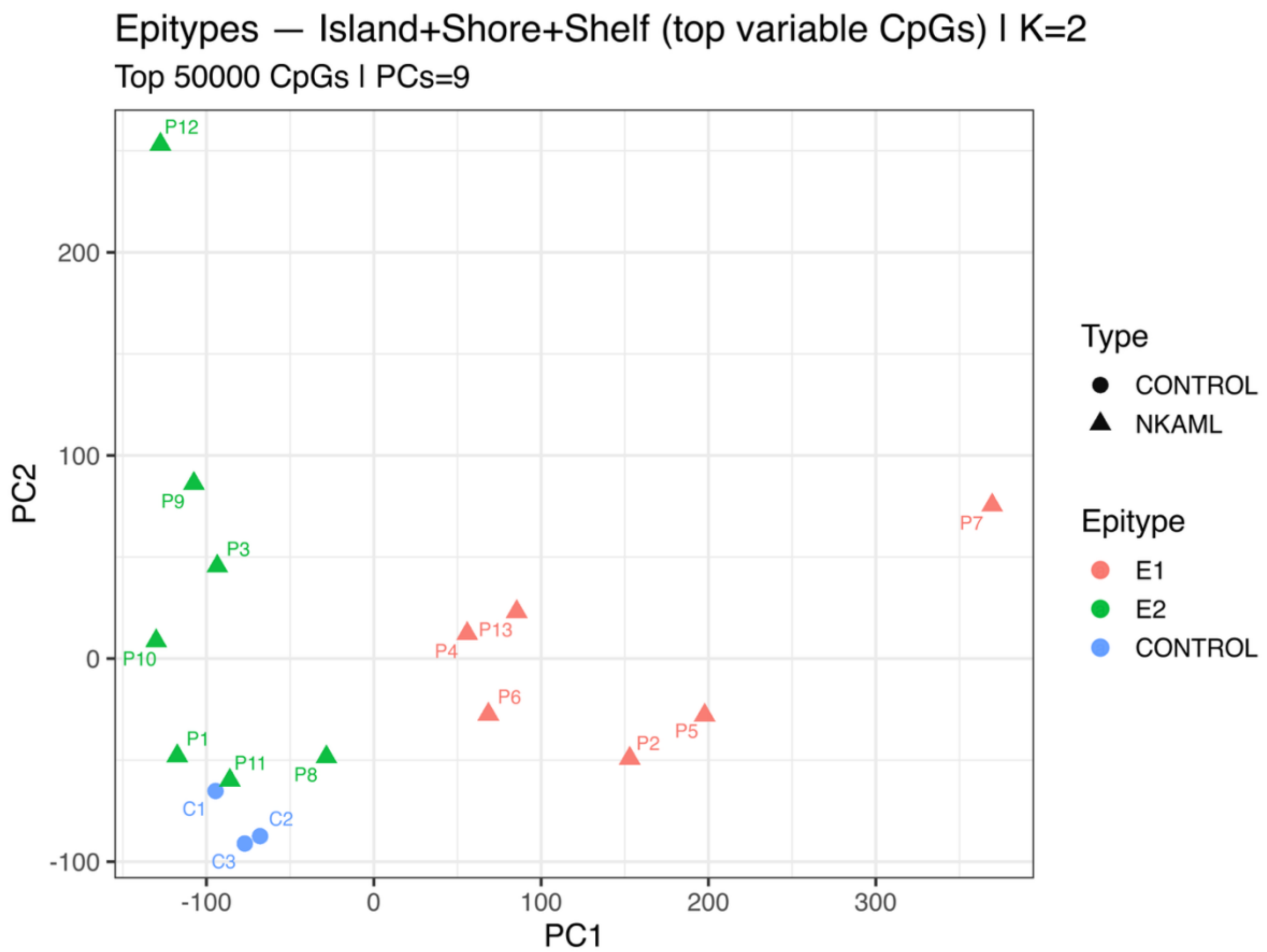
Epitypes — Island+Shore+Shelf (top variable CpGs) | K=2
Top 50000 CpGs | PCs=9
PC2
PC1
200
100
0
-100
-100
0
100
200
300
P12
P9
P3
P10
P1
P11
P8
C1
C2
C3
P13
P4
P6
P2
P5
P7
Type
CONTROL
NKAML
Epitype
E1
E2
CONTROL

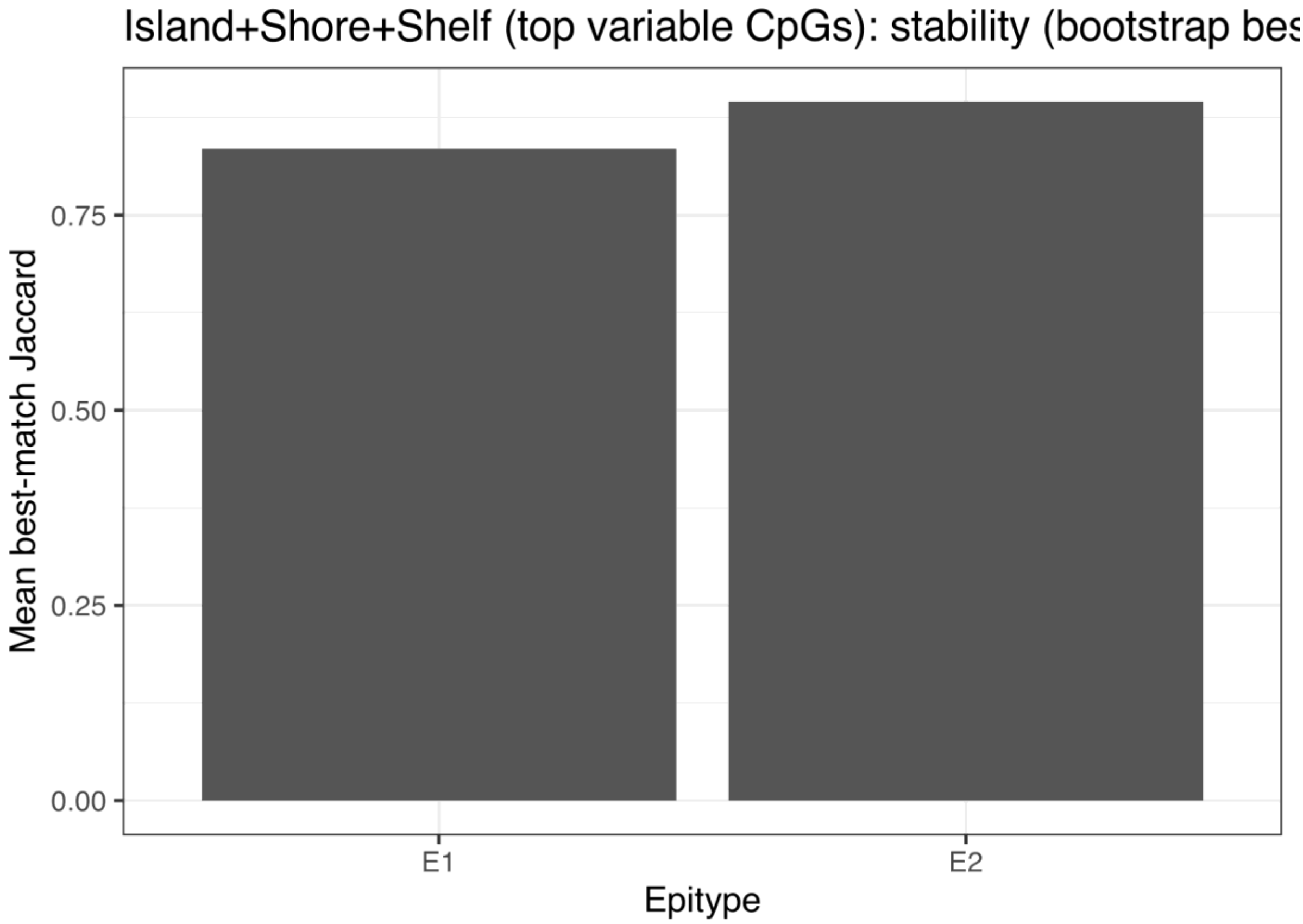

FeatureSet	Covariate	Pvalue
Wholearray	Array	0.505857105857102
Wholearray	Slide	0.95027195027195
Promoter	Array	0.944055944055944
Promoter	Slide	0.592074592074592
CGI	Array	0.944055944055944
CGI	Slide	0.592074592074592
